# Extracellular matrix remodeling in animal models of anthracycline-induced cardiomyopathy: a meta-analysis

**DOI:** 10.1007/s00109-021-02098-8

**Published:** 2021-05-29

**Authors:** Jan M. Leerink, Mabel van de Ruit, Elizabeth A.M. Feijen, Leontien C.M. Kremer, Annelies M.C. Mavinkurve-Groothuis, Yigal M. Pinto, Esther E. Creemers, Wouter E.M. Kok

**Affiliations:** 1 Department of Clinical and Experimental Cardiology, Amsterdam University Medical Centers, University of Amsterdam, Meibergdreef 9, 1105 AZ Amsterdam, The Netherlands; 2 Princess Máxima Center for Pediatric Oncology, Utrecht, The Netherlands

**Keywords:** Anthracyclines, Cardiotoxicity, Extracellular matrix remodeling, Fibrosis, Animals, Systematic review

## Abstract

As in other cardiomyopathies, extracellular matrix (ECM) remodeling plays an important role in anthracycline-induced cardiomyopathy. To understand the pattern and timing of ECM remodeling pathways, we conducted a systematic review in which we describe protein and mRNA markers for ECM remodeling that are differentially expressed in the hearts of animals with anthracycline-induced cardiomyopathy. We included 68 studies in mice, rats, rabbits, and pigs with follow-up of 0.1–8.2 human equivalent years after anthracycline administration. Using meta-analysis, we found 29 proteins and 11 mRNAs that were differentially expressed in anthracycline-induced cardiomyopathy compared to controls. Collagens, matrix metalloproteinases (MMPs), inflammation markers, transforming growth factor ß signaling markers, and markers for cardiac hypertrophy were upregulated, whereas the protein kinase B (AKT) pro-survival pathway was downregulated. Their expression patterns over time from single time point studies were studied with meta-regression using human equivalent years as the time scale. Connective tissue growth factor showed an early peak in expression but remained upregulated at all studied time points. Brain natriuretic peptide (BNP) and MMP9 protein levels increased in studies with longer follow-up. Significant associations were found for higher atrial natriuretic peptide with interstitial fibrosis and for higher BNP and MMP2 protein levels with left ventricular systolic function.

**Supplementary Information:**

The online version contains supplementary material available at 10.1007/s00109-021-02098-8.

## Introduction

Anthracyclines are a class of chemotherapeutic agents used to treat various types of cancer and they have contributed to a significant improvement in survival of cancer patients [1]. However, anthracycline treatment is associated with left ventricular dysfunction and heart failure in a dose-dependent manner and can occur up to decades after exposure [2].

Understanding the harmful mechanisms leading to anthracycline-induced cardiomyopathy and their timing in the transition to heart failure is critical to develop strategies for early detection, prevention, and treatment. While the exact mechanism remains unclear, multiple processes have been identified to be involved in anthracycline-induced cardiotoxicity [3, 4]. A major role is attributed to inhibition of topoisomerase 2 activity, a nuclear enzyme required for DNA transcription and replication, which is followed by the formation of reactive oxygen species, mitochondrial dysfunction, and apoptosis of cardiomyocytes [5].

As a central theme in cardiac remodeling and response to excess loading or injury, a hypertrophic response occurs within cardiomyocytes, but also in cardiac fibroblasts, which are ubiquitously present in the heart [6]. Excess loading conditions may trigger transforming growth factor ß (TGFß)–induced fibroblast activation, which results in excessive production of extracellular matrix (ECM) components and ventricular dysfunction [7]. Irrespective of its cause, cardiomyocyte injury by itself triggers an inflammatory reaction, which also induces fibroblast activation [6–9].. In anthracycline-induced cardiomyopathy, both inflammatory and adverse ECM remodeling processes are present [4, 10–14]. Several transcriptomic analysis in animal and in vitro studies have reported differential expression in genes related to the innate immune system, TGFß signaling, and collagen turnover [10, 15–18]. Understanding the pathways and timing of ECM remodeling in anthracycline-induced cardiomyopathy is urgently needed to identify potential targets for treatment and blood markers that may be used during surveillance. In this systematic review, we had 3 objectives: (1) to find which ECM remodeling markers are significantly upregulated or downregulated in the hearts of animals with anthracycline-induced cardiomyopathy compared to control animals; (2) to delineate possible temporal expression patterns of ECM remodeling markers, and (3) to find associations of ECM remodeling marker levels with interstitial fibrosis, left ventricular systolic function and/or apoptosis.

## Methods

This systematic review adhered to the Preferred Reporting Items for Systematic Reviews and Meta-Analyses (PRISMA) guidelines [19]. The protocol was registered in PROSPERO (ID: CRD42020161338), an international database of systematic review protocols.

### Search strategy, eligibility criteria, and risk of bias assessment

PubMed and EMBASE were systematically searched for studies measuring markers for ECM remodeling in animals with anthracycline-induced cardiomyopathy (search strategy in Supplementary Material). The reference list of included articles was screened for additional studies. Two authors independently screened studies and included studies with in vivo administration of anthracyclines and with analysis of ECM proteins or mRNAs in heart tissue after at least 2 doses of anthracyclines, as this more closely reflects the situation in humans. Excluded were studies without anthracycline exposure, in vitro studies, studies with another concomitant cardiotoxic intervention, conference abstracts, studies not describing ECM remodeling markers, and studies written in other languages than English, French, or German. Discrepancies between the reviewers were discussed and resolved in group. Risk of bias was assessed with the systematic review center for laboratory animal experimentation risk of bias checklist by two authors [20].

### Data extraction

Data was extracted with a predefined form. Data only available in graphs was extracted with Adobe Acrobat Pro. Extracted were the number of animals, age at start of anthracyclines, time of sampling after the first anthracyclines dose, the anthracycline derivative, and the cumulative dose in mg/kg. The mean and standard deviation of ECM remodeling markers, left ventricular ejection fraction (LVEF) or fractional shortening (FS), interstitial fibrosis area, and cardiomyocyte apoptosis were extracted. If the standard deviation was not reported, it was calculated from the standardized error of the mean using the formula: standard deviation=standardized error of the mean*√(number of animals).

### Age equivalency and follow-up comparison between animal species

To compare follow-up time after first anthracycline injection between animal species, we converted animal age and follow-up duration to human equivalent years based on the maximum life span of each animal species obtained from the AnAge longevity database (mice 4.0 years, rats 4.2 years, rabbits 9 years, pigs 27 years, humans 90 years) (https://genomics.senescence.info/species/). Separate analyses were performed for small animals (mice, rats, rabbits) and pigs due to the differences in body mass and life expectancy.

### Objective 1: ECM remodeling markers in anthracycline-induced cardiomyopathy animals compared to control animals

We defined markers for ECM remodeling as proteins or mRNAs implicated in ECM remodeling as described by the authors of each study or as described in the literature. For every ECM remodeling marker described in each study, we calculated the ratio of the means (ROM) by dividing mean expression in cardiomyopathy animals by mean expression in control animals. A ROM >1 indicates a higher mean expression in anthracycline-induced cardiomyopathy whereas a ROM <1 indicates higher expression in controls [21]. We used a random effects meta-analysis to pool the ROM of each marker across studies and considered markers with a ROM >1.2 or <0.83 and a p-value <0.05 as significantly up- or downregulated, respectively. We used the Hartung and Knapp method to estimate p-values and 95% confidence intervals, as it has been shown to be more accurate in situations with moderate to substantial inter-study heterogeneity [22]. For presentation, we classified proteins and mRNAs into pathways as described in the literature and Reactome (reactome.org). Protein quantity (measured with Western blot) and gelatinase activity (measured with zymography) of MMP2 and MMP9 were analyzed together to increase power since we did not observe differences in temporal trends when we analyzed them separately. mRNA expression of MMPs was measured in studies with quantitative polymerase chain reaction.

### Objective 2: Temporal expression patterns of ECM remodeling markers

We used meta-regression to study temporal patterns in ECM marker levels after anthracycline injection. Meta-regression is a method to study the effect of an exploratory variable (time in our study) on the effect estimate of each study (ROM in our study) [21]. In this analysis, we only included ECM markers measured at ≥5 unique time points, either within the same study or in different studies that measured the same marker.

### Objective 3: Association of ECM remodeling markers with fibrosis, LV function, and apoptosis

We also used meta-regression to study the association of ECM marker levels with (1) myocardial interstitial fibrosis area (quantified with the standardized mean difference (SMD)), (2) LV systolic function (LVEF or FS) quantified with the SMD), and (3) cardiomyocyte apoptosis detected with terminal deoxynucleotidyl transferase dUTP nick end labeling (TUNEL, quantified with the SMD). The SMD represents the difference in means between cardiomyopathy and control animals divided by the standard deviation, which is useful when measurements are on a different scale [21]. Only markers with ≥5 measurements were analyzed. Meta-analysis and meta-regression were performed in R studio version 3.6.1 using the “metafor” package. A two-sided p-value <0.05 was considered statistically significant. P-values were not corrected for multiple testing in this hypothesis-generating study.

### Assessment of inter-study heterogeneity

Heterogeneity was quantified with the I^2^ statistic. An I^2^ between 0 and 25% reflects very low heterogeneity, 25–50% reflects low heterogeneity, 50–75% reflects moderate heterogeneity, and an I^2^ >75% reflects substantial heterogeneity [21]. As substantial heterogeneity was expected in animal studies and this review is hypothesis generating, we also describe the concordance in the direction of the effect (i.e., upregulation or downregulation in all studies).

## Results

### Overview of included studies in the systematic review

**Fig. 1:**
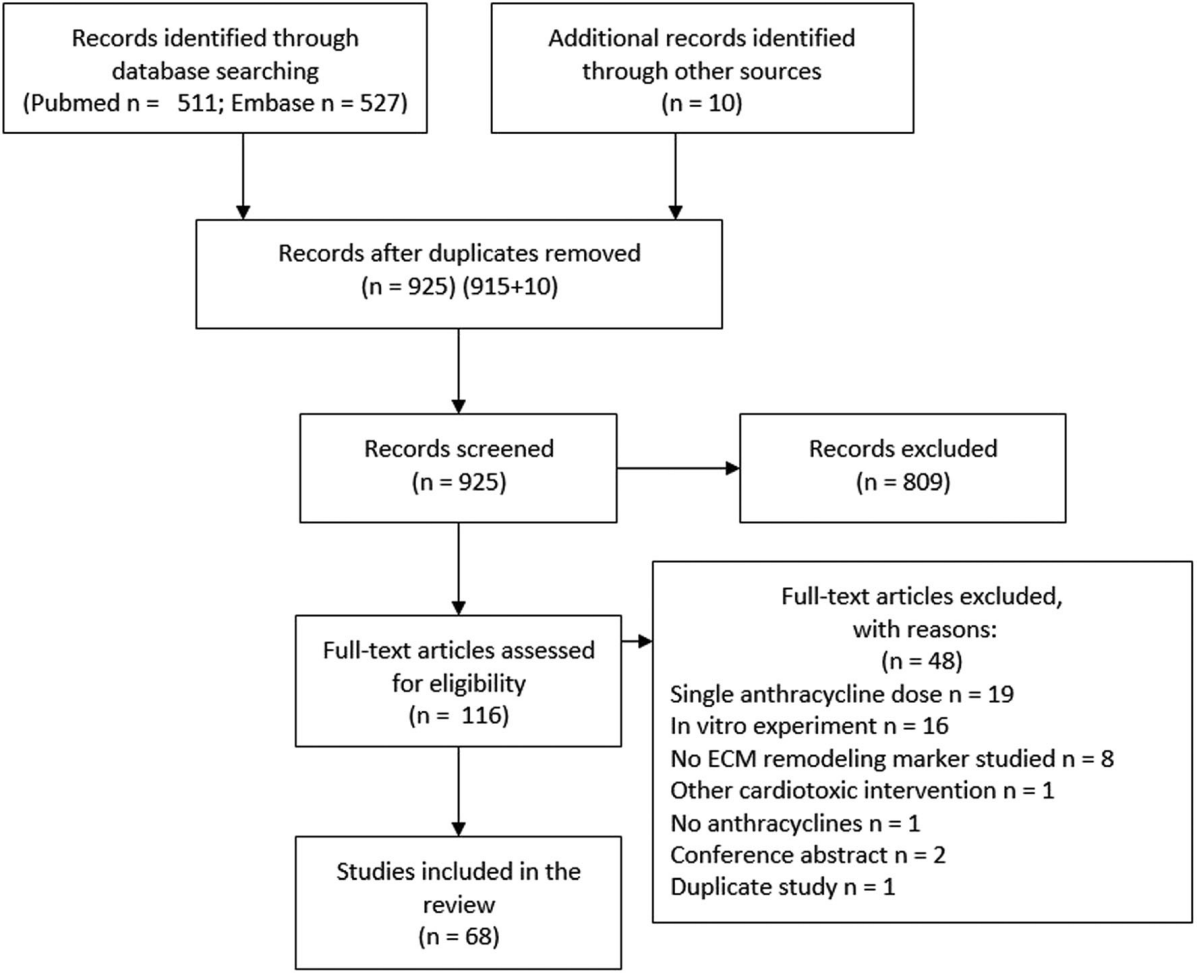
PRISMA flowchart of study inclusion in the systematic review. ECM=extracellular matrix

Using the search terms indicated in the supplementary material, we identified 915 original studies by searching PubMed and Embase. In addition, we identified 10 studies from expert knowledge and by reviewing the references of the studies (Fig. 1). After exclusion of 809 studies based on title and abstract, we screened the full text of 116 studies. Main exclusion reasons were a single anthracycline dose (n = 19), in vitro experiments in cell lines (n = 16), and no marker for ECM remodeling studied (n = 8). We included in total 68 studies in mice (19 studies, 312 animals), rats (40 studies, 752 animals), rabbits (6 studies, 96 animals), and pigs (3 studies, 43 animals ) in the systematic review. Study characteristics are shown in Table S1 and are summarized in Table 1. The cumulative anthracycline dose ranged from 2.3 to 40.0 mg/kg with a dose of 15.0 mg/kg most commonly used. Protein or mRNA levels were measured in heart tissue at 2 days up to 20 weeks after the first anthracycline injection corresponding to a human equivalent follow-up of 0.1 to 8.2 years. In the majority of studies, cardiomyopathy severity was assessed with cardiac function measurements (LVEF or FS). Histologic examination revealed interstitial fibrosis, cytoplasmic vacuolization, and loss of myofibrils in most of the studies (Table S2) [23].

**Table 1:** Summary of the included studies stratified by animal species (characteristics per study are shown in Table S1)

Characteristic	Mice 19 studies	Rats 40 studies	Rabbits 6 studies	Pigs 3 studies
Number of animals	312	752	96	43
Median anthracycline dose, mg/kg (IQR)	15.0 (12.0, 24.0)	15.0 (12.0, 17.1)	30.0 (21.0, 30.0)	2.9 (2.6, 3.5)
Median weeks after injection (range)	5.0 (1.0, 18.0)	4.0 (0.3, 20.0)	10.0 (8.0, 20.0)	8.6 (4.3, 16)
Human equivalent follow-up in years, median (range)	2.2 (0.4, 7.8)	1.6 (0.1, 8.2)	1.9 (1.5, 3.8)	0.5 (0.3, 1.0)
LVEF and/or FS measurement	14 studies	18 studies	3 studies	3 studies
Fibrosis area measurement	13 studies	21 studies	5 studies	1 studies
Proteins/mRNAs measured	Proteins n = 44, mRNAs n = 19			

*Abbreviations*: *IQR* inter quartile range, *LVEF* left ventricular ejection fraction, *FS* fractional shortening

### Objective 1: ECM remodeling marker expression in anthracycline-induced cardiomyopathy compared to control hearts

**Fig. 2:**
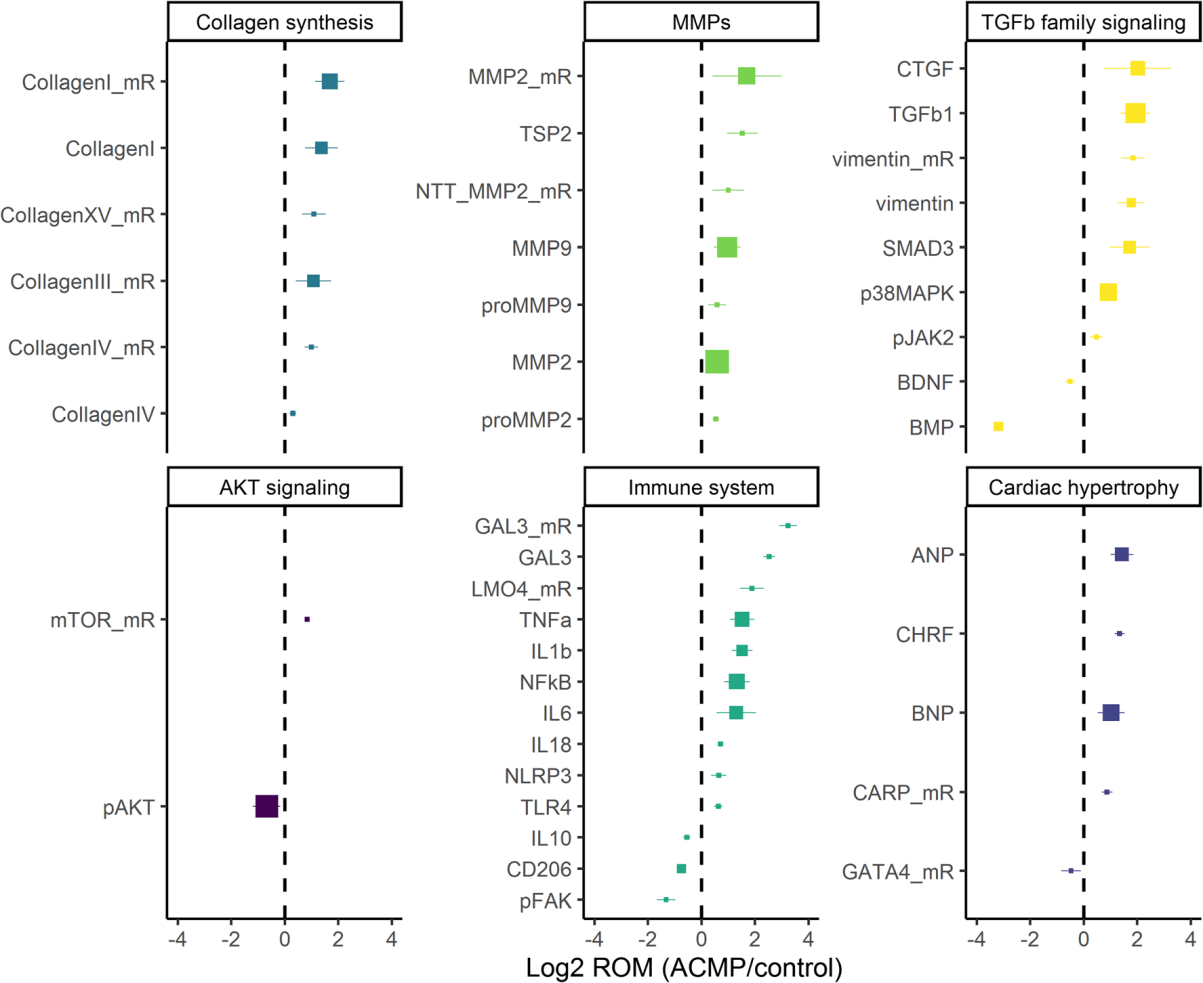
Differentially expressed proteins and mRNAs from the random effects meta-analysis of studies in mice, rats, and rabbits with anthracycline-induced cardiomyopathy (ACMP). The x-axis indicates the log2 ratio of the means (ROM) from the random effects meta-analysis in ACMP compared to controls. Differentially expressed proteins and mRNAs (mR) with a ROM >1.2 or <0.83 and p-value <0.05 are shown. The size of each square is proportional with the number of studies in which the protein or mRNA was measured. Lines indicate the 95% confidence interval of the log2 ROM. MMP=matrix metalloproteinase, NTT=N-terminal truncated, TSP=thrombospondin, CTGF=connective tissue growth factor, TGFb1=transforming growth factor beta 1, SMAD=mothers against decapentaplegic homolog, p38 MAPK (p38 mitogen-activated protein kinases), pJAK2=phosphorylated janus kinase 2, BDNF=brain-derived neurotrophic factor, BMP=bone morphogenetic protein, mTOR=mechanistic target of rapamycin kinase, pAKT=phosphorylated protein kinase B, GAL3=galectin 3, LMO4=LIM domain transcription factor, TNFa=tumor necrosis factor alpha, IL=interleukin, NFkB=nuclear factor kappa B, NLRP3=NLR family pyrin domain containing 3, TLR4=toll-like receptor 4, CD206=cluster of differentiation factor 206/mannose receptor on macrophages, pFAK=phosphorylated focal adhesion kinase, ANP=atrial natriuretic peptide, BNP=brain natriuretic peptide, CHRF=cardiac hypertrophy-related factor, CARP=cardiac adriamycin-responsive protein, GATA4=GATA binding protein 4

Results of the meta-analysis in 65 studies in mice, rats, and rabbits are shown in Fig. 2. We identified 30 proteins and 12 mRNAs that were significantly up- or downregulated in anthracycline-induced cardiomyopathy as compared to controls (random effect p-value <0.05 and ROM >1.20 or <0.83). We classified them in 6 pathways: (1) collagen synthesis, (2) matrix metalloproteinases, (3) transforming growth factor ß (TGFß) signaling, (4) protein kinase B (AKT) signaling, (5) immune system, and (6) cardiac hypertrophy. Markers upregulated more than 3-fold were TGFß1 (ROM 3.80, n = 13 studies), CTGF (ROM 4.04, n = 6), SMAD3 (ROM 3.27, n = 7), MMP2 mRNA (ROM 3.23, n = 9), collagen I mRNA (ROM 3.22, n = 7), and GAL3 (mRNA ROM 9.46, protein ROM 5.78, n = 1) (Fig. 2). Markers of interest that were upregulated less than 3-fold were MMP9 (ROM 1.94, n = 13), MMP2 (ROM 1.50, n = 19), TNFα (ROM 2.88, n = 6), and IL6 (ROM 2.46, n = 5 studies, p = 0.03).

Due to the limited number of studies in pigs (3 studies), we report results descriptively. Goetzenich et al. studied matrix metalloproteinases at a human equivalent follow-up of ±0.3 years in pigs (mean LVEF 38%) treated with 5–7 intracoronary doses of 25 mg doxorubicin (±2.9 mg/kg) [24]. MMP2 and MMP1 activities, measured with fluorogenic assay, were increased 5 to 8-fold compared to controls, respectively. mRNA levels of MMP1 (4.9-fold), MMP2 (5.4-fold), MMP9 (4.7-fold), and membrane type-1 MMP (3.2-fold) were also significantly increased. The mRNA expression of MMP3, TIMP1, and collagen 1 was not significantly altered in this study [24].

Gyongyosi et al. performed RNA sequencing in pigs with less severe cardiomyopathy (mean LVEF 45%, cumulative doxorubicin dose 180 mg/m^2^, ±4.1 mg/kg) and with a slightly longer follow-up of ±0.6 human equivalent years. In transcriptomic analysis, they showed differential expression of genes in the TGFß signaling pathway, ECM genes (fibroblast activation markers osteonectin and tenascin-c), DNA damage genes, collagen synthesis genes, and growth factors. TIMP2 mRNA expression was significantly downregulated but mRNA levels of MMPs were not different in pigs with anthracycline-induced cardiomyopathy compared to controls [18].

Galán-Arriola et al. studied pigs with doxorubicin-induced cardiomyopathy (mean LVEF 32.5%, cumulative dose 2.25 mg/kg) and found a significant increase compared to healthy controls in mitochondrial fragmentation and in interstitial fibrosis area at ±1.0 human equivalent years [25].

### Objective 2: Temporal changes in ECM remodeling markers

**Fig. 3:**
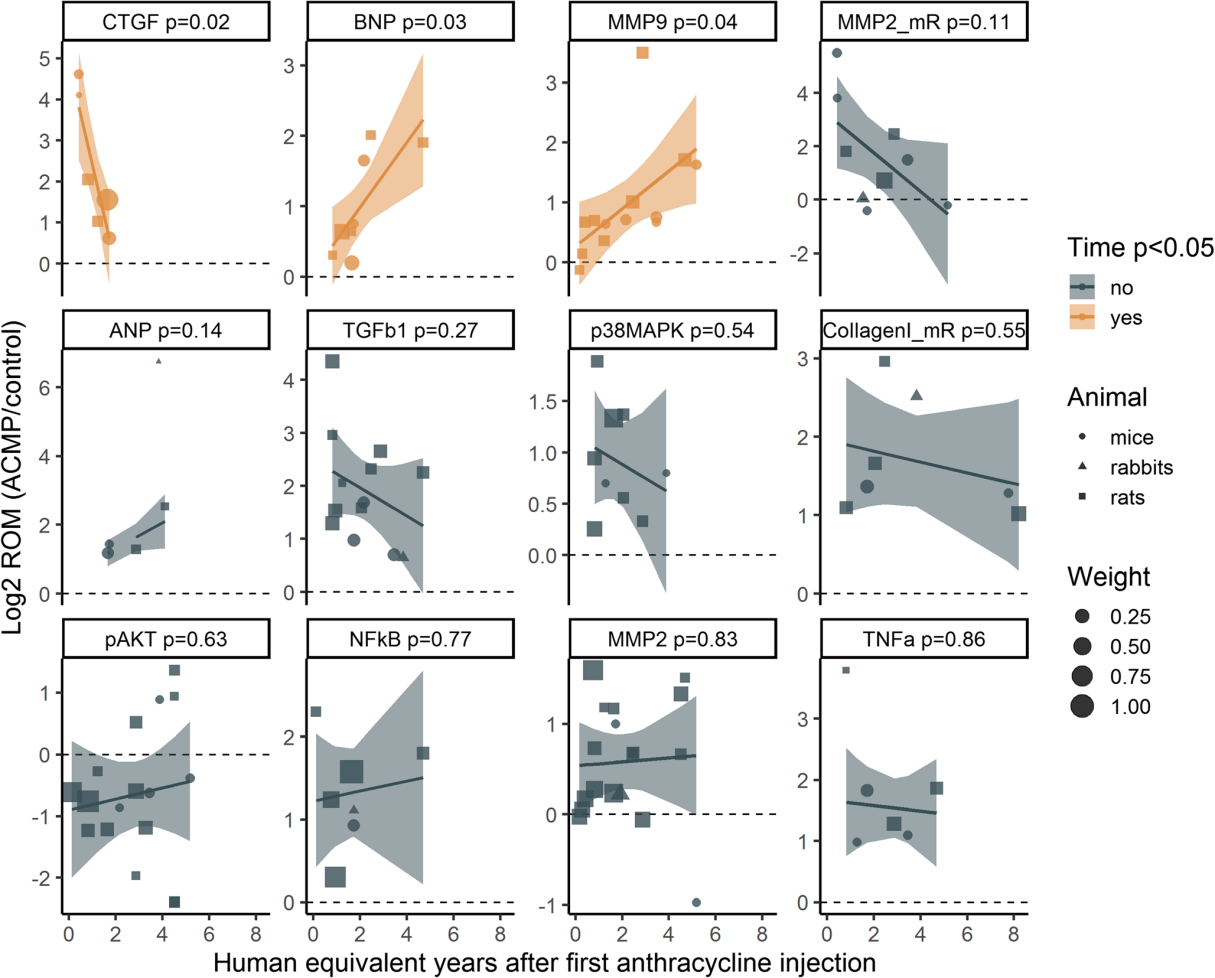
Temporal expression patterns of extracellular matrix-related proteins in mouse, rat, and rabbit models of anthracycline-induced cardiomyopathy (ACMP). Every dot represents one study where the size is proportional to the weight in the meta-regression. Lines and ribbons represent the meta-regression fit and 95% confidence interval. Proteins significantly association with time after first anthracycline injection in the meta-regression were connective tissue growth factor (CTGF, negative association, p = 0.02), brain natriuretic peptide (BNP, positive association, p = 0.03) and matrix metalloproteinase 9 (MMP9, positive association, p = 0.04). ANP=atrial natriuretic peptide, MMP2=matrix metalloproteinase 2, mR=mRNA, NF-κB=nuclear factor-κB, TGFß=transforming growth factor beta, TNFα=tumor necrosis factor alpha, pAKT=phosphorylated protein kinase B, p38 MAPK=p38 mitogen-activated protein kinases

We used meta-regression to study temporal expression trends of proteins and mRNAs that were differentially expressed in our meta-analysis and were measured in at least 5 of the included studies. Results are presented in Table S3 and illustrated in Fig. 3. We found 3 proteins of which expression in anthracycline-induced cardiomyopathy compared to control animals (ROM) changed significantly in studies with longer follow-up after anthracyclines. Connective tissue growth factor (CTGF) protein was measured in studies performed at 0.4–1.7 human equivalent years after anthracycline administration. CTGF levels were higher in anthracycline-induced cardiomyopathy compared to controls in all studies with a peak at 0.4 human equivalent years after anthracycline administration and a decrease in studies with longer follow-up (1.70 lower ROM per year, meta-regression p = 0.03). MMP9 protein was studied at 0.2–5.2 years and levels were higher in anthracycline-induced cardiomyopathy compared to control animals in studies with longer follow-up after anthracycline administration (0.32 higher ROM per year, p = 0.04). Brain natriuretic peptide (BNP) was studied at 0.8–4.7 years and levels were higher in anthracycline-induced cardiomyopathy compared to control animals in studies with longer follow-up after anthracycline administration (0.32 higher ROM per year, p = 0.04).

We observed consistent upregulation at ≥5 time points without a significant temporal trend in TGFß1 protein (0.8–4.7 human equivalent years), p38 mitogen-activated protein kinase (p38 MAPK) protein (0.8–3.9 years), tumor necrosis factor (TNFα) protein (0.8–4.7 years), nuclear factor-κB (NF-κB) protein (0.1–4.7 years), atrial natriuretic peptide (ANP) protein (0.4–4.1 years), and collagen I mRNA (0.8–8.2 years) (Fig. 3, Table S3).

### Objective 3: Association of ECM remodeling markers with interstitial fibrosis, LV function, and cardiomyocyte apoptosis

**Fig. 4:**
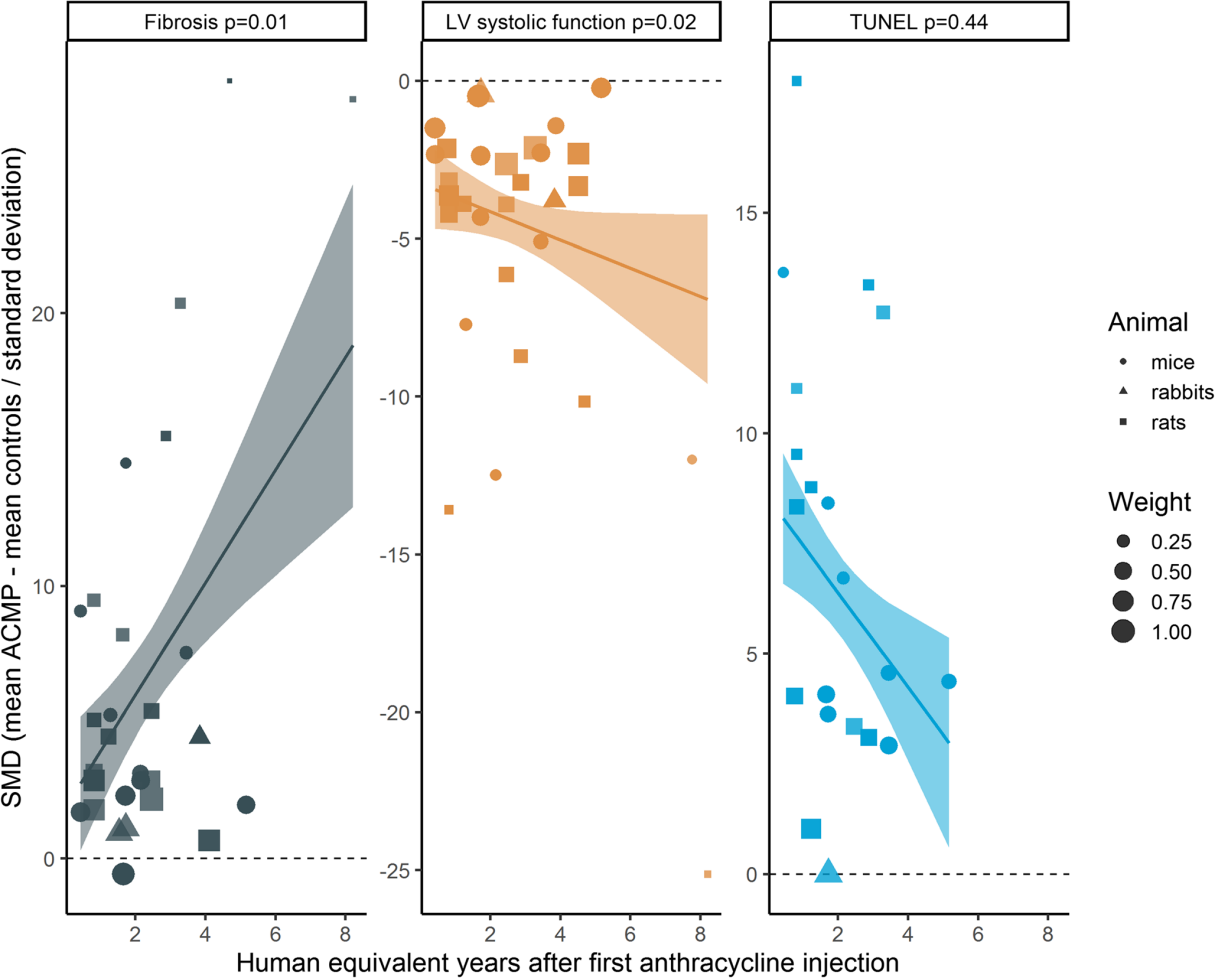
Relationship between interstitial fibrosis area (A), systolic function (B), apoptosis (C), and time after first anthracycline injection in individual studies in mice, rats, and rabbits. Every dot represents one study where the size is proportional to the weight in the meta-regression. Lines and ribbons represent the meta-regression fit and 95% confidence interval, respectively. Interstitial fibrosis area, left ventricular (LV) systolic function (LV ejection fraction (LVEF) or fractional shortening (FS)), and apoptosis (terminal deoxynucleotidyl transferase dUTP nick end labeling (TUNEL)) were expressed as a standardized mean difference (SMD) between anthracycline-induced cardiomyopathy (ACMP) and control animals (calculated as the difference in means between ACMP animals and control animals divided by the standard deviation)

We studied associations of interstitial fibrosis area, LV systolic function, and cardiomyocyte apoptosis with time after anthracycline administration (Fig. 4) and with protein or mRNA levels (Table S3) using meta-regression of studies performed in mice, rats, and rabbits.

Interstitial fibrosis area, investigated with Sirius red stainings in 38 studies, was more pronounced in anthracycline-induced cardiomyopathy as compared to control animals (SMD 5.70, p < 0.0001) and increased in studies with longer follow-up (1.71 increase in SMD per human equivalent year, p = 0.01) (Fig. 4). Two studies, performed in rats and mice, reported a significant increase in interstitial fibrosis area as early as 0.4 human equivalent years after anthracyclines [26, 27]. Higher ANP protein levels were associated with a larger interstitial fibrosis area (0.76 higher ROM per 1 SMD increase in interstitial fibrosis area, p = 0.01) (Table S3). The expression of TGFß1, CTGF, MMPs, NFkB, and TNFα was not significantly associated with interstitial fibrosis area.

LV systolic function, measured with LVEF or FS in 40 studies, was worse in anthracycline-induced cardiomyopathy compared to control animals (SMD − 3.65, p < 0.0001) and lower LV systolic function was observed in studies with longer follow-up (− 0.77 SMD decrease per 1 human equivalent year, p = 0.02) (Fig. 4). LV measurements were performed in anesthetized animals in 36 studies and in 4 studies this was unclear. Higher MMP2 protein (p = 0.02) and BNP protein (p = 0.04) levels were associated with worse LV systolic function (Table S3).

Cardiomyocyte apoptosis, measured with TUNEL in 28 studies, was higher in anthracycline-induced cardiomyopathy compared to control animals (SMD 6.21, p < 0.0001). This increase in apoptosis as measured with TUNEL was confirmed in 9 studies using caspase 3 (SMD 9.03, p = 0.01). A decreasing trend in apoptosis was observed in studies with a longer follow-up after first anthracycline injection, although not statistically significant (p = 0.44, Fig. 4). The study with the longest follow-up after anthracycline administration (56 days, 3.5 human equivalent years) in mice treated with a cumulative dose of 12 mg/kg still demonstrated apoptosis of cardiomyocytes and vascular smooth muscle cells [28]. In meta-regression, higher mRNA expression of MMP2 (p = 0.02) and higher protein levels of TGFß1 (p = 0.03) were significantly associated with more cardiomyocyte apoptosis (Table S3).

### Inter-study heterogeneity in marker expression

Significant heterogeneity in marker expression values was present between studies in most of the markers pooled in the meta-analysis (I^2^ > 75%). However, the direction of the marker expression was the same in all studies except for MMP2 (downregulation in one study that used a higher cumulative dose of 24 mg/kg [29]) and phosphorylated AKT (upregulation in 3 studies [13, 30, 31]). We did not observe differences in anthracycline dose and/or animal species that could explain heterogeneity in AKT expression.

### Risk of bias assessment

Risk of bias was unknown in the majority of the included studies (Table S4). Random group allocation was performed in 34/68 studies and blinding of the outcome assessor and investigators was reported in 15 of the included studies. Attrition bias might have been present in 17 studies where animal dropouts were not explained sufficiently (Table S4).

## Discussion

To better understand the association of ECM remodeling with anthracycline-induced cardiomyopathy development, we explored the presence and temporal patterns in protein and mRNA markers for ECM remodeling in 68 animal studies. In our meta-analysis, we demonstrate that collagens, matrix metalloproteinases MMP2 and MMP9, immune system markers and markers in the TGFß signaling, and cardiac hypertrophy pathway are upregulated in anthracycline-induced cardiomyopathy whereas the AKT pro-survival pathway is downregulated. Furthermore, by using meta-regression, we show temporal changes in markers for TGFß-induced fibroblast activation (CTGF protein), cardiac hypertrophy/wall stress (BNP protein), and MMP9. In addition, with this meta-regression technique, we were also markers for ECM remodeling that were associated with worse LV systolic function (BNP and MMP2 protein) and with more interstitial fibrosis (ANP protein). In the following sections, we discuss the markers identified in our systematic review per pathway. In the final section, we summarize the overall temporal ECM remodeling patterns in anthracycline-induced cardiomyopathy.

### TGFß signaling and collagen synthesis

TGFß1 is the key initiator of fibroblast-myofibroblast conversion and collagen synthesis in the heart [7]. In our meta-analysis, TGFß1 and one of its signal transducers (SMAD3) were consistently upregulated two to fourfold in the hearts of animals with anthracycline-induced cardiomyopathy from 0.8 to 4.7 human equivalent years after anthracycline administration, suggesting pro-fibrotic signaling is present at both early and late stages. A downstream target of the canonical TGFß1-SMAD pathway, CTGF, was upregulated in all studies included in our meta-analysis, with an early peak in studies performed within 1 human equivalent years after anthracycline administration [7]. A pressure overload animal model also demonstrated this early peak upregulation in CTGF in the heart, followed by a later reduced but steady upregulation [32]. In addition, the non-canonical p38 MAPK pathway was consistently upregulated in our review, which is also a critical regulator of fibrotic remodeling [33]. Fibroblast activation markers, such as osteonectin mRNA and tenascin-c mRNA, were upregulated in one of the two reviewed studies in pigs [18]. Finally, as a likely result of the above described pro-fibrotic signaling, we observed upregulation of collagen type I (protein) and III (mRNA) and an increase in interstitial fibrosis area at longer follow-up durations after anthracyclines.

### Matrix metalloproteinases

Matrix metalloproteinases (MMPs) are present in the normal heart in an inactive form. Activation of MMPs, especially the gelatinases MMP-2 and MMP-9, is associated with adverse remodeling and LV dilatation in heart failure patients, and precedes LV dysfunction in animal models with tachycardiomyopathy, suggesting that they are early markers of cardiomyopathy [34]. MMP2 and MMP9 are expressed and secreted by cardiac fibroblasts, cardiomyocytes, endothelial cells, and immune cells [35]. During secretion, a substantial fraction of MMP2, and likely also MMP9, remains intracellular where it can be activated [36]. MMP2 is activated by oxidative stress in cardiomyocytes, in part due to upregulating N-terminal-truncated intracellular MMP2, which explains the acute presence and activity of MMP2 in anthracycline-induced cardiomyopathy [37, 38]. Transcription and activation of MMP2 can also be enhanced by the innate immune system, including NFkB-signaling [39, 40]. Interestingly, this innate immune system activation is also triggered by N-terminal truncated MMP2 [36]. MMP2 is most well-known for proteolizing ECM proteins but is also active inside cardiomyocytes where it cleaves sarcomeric proteins [38].

In our meta-regression, MMP2 and MMP9 were upregulated in anthracycline-induced cardiomyopathy compared to controls and MMP9 levels were higher in studies with longer follow-up after anthracyclines. In addition, a strongly increased MMP9 mRNA expression was found in one of the included studies in pigs [24]. To localize MMPs, studies demonstrated MMP2 activity in sarcomeres and mitochondria of cardiomyocytes [38] and next to areas with fibrosis [24, 41], while MMP1 activity (a collagenase) was observed mainly surrounding blood vessels and surviving cardiomyocytes [41]. We did not find studies that localized MMP9, but their source is assumed to be mainly from macrophages [28].

MMP activity should be interpreted together with the activity from their inhibitors (TIMPs) [34]. In our systematic review, TIMP2 mRNA was downregulated in one study in pigs from Gyongyosi et al. [18] TIMPs (types 1, 2, 3, and 4) were not differentially expressed in our meta-analysis of 6 studies in mice, rats, and rabbits.

The matricellular protein thrombospondin-2 (TSP2), mainly expressed by fibroblasts, is known to inhibit the proteolytic activity of MMP2 by binding to active MMP2 [42]. In one of the studies included in our systematic review, TSP2 was upregulated early after doxorubicin treatment of wild-type mice compared to control mice while MMP2 was not upregulated [29]. In the same study, TSP2 knock-down mice treated with doxorubicin showed enhanced MMP2 activity and ECM disruption compared to doxorubicin-treated wild-type mice [29]. This suggests that TSP2 controls MMP2 activity.

### Markers for inflammation

In response to cardiomyocyte injury, the innate immune system is activated with release of pro-inflammatory cytokines and chemokines that promote immune cell infiltration and initiate pathological LV remodeling [6]. Toll-like receptors, expressed on macrophages and dendritic cells, act as sensors for doxorubicin-induced cell death (through recognition of damage-associated molecular pattern molecules (DAMPs)) and initiate release of the inflammatory cytokines (e.g., TNFα) and chemokines (e.g., C-C motif chemokine ligands) [43–45]. In the setting of chronic inflammation, these pro-inflammatory cytokines can induce pathological ECM remodeling in animal models [9]. For example, in mice that selectively overexpressed TNFα, MMPs were activated in the initial stages of inflammation resulting in ECM degradation and LV dilatation, while prolonged inflammation promoted mast cell–mediated TGFß signaling and collagen synthesis [9, 46, 47]. The NF-κB protein complex, which is present in almost every cell type, is involved in the innate immune system and regulates cell survival and cytokine production [48]. NF-κB and TNFα were consistently upregulated in all studies included in our systematic review, which suggests (chronic) inflammation in anthracycline-induced cardiomyopathy. While NF-κB has a cardioprotective role during acute myocardial injury by preventing cardiomyocyte apoptosis, persistent activation of NF-κB is maladaptive by inducing production of the inflammatory cytokines TNFα, IL6, and IL1ß [48]. Whether the persistent increase in inflammation markers we observed is the result of ongoing release of DAMPs by injured cardiomyocytes or that other processes also contribute, such as the release of pro-inflammatory ECM fragments during matrix degradation by MMPs [9], is unknown.

### Cardiac hypertrophy and wall stress

Natriuretic peptides are established markers for ventricular wall stress and cardiac hypertrophy and have anti-fibrotic properties [32, 49]. Not surprisingly, in our review, higher BNP levels were present in studies with longer follow-up after anthracyclines and higher BNP levels were associated with lower LV function. In addition, higher ANP levels were associated with more interstitial fibrosis, in agreement with previous studies [49, 50].

### AKT signaling

AKT signaling regulates cardiac hypertrophy, angiogenesis, and glucose metabolism and promotes survival [51]. It has been shown that in situations with increased cardiac stress, short-term activation of AKT prevents cardiomyocyte apoptosis and promotes physiological hypertrophy, while long-term activation induces pathological hypertrophy and ECM remodeling [52].

In our review, phosphorylated AKT was downregulated in most of the included studies. However, two studies in rats included in our review reported upregulations in phosphorylated AKT [13, 30]. These seem to be exceptions, however, and overall downregulated AKT levels were seen, which suggests both impaired cardiomyocyte survival signaling in anthracycline-induced cardiomyopathy, as well as a potential failed protective effect against pathological ECM remodeling.

### Limitations

Some limitations should be considered. First, heterogeneity in marker expression was significant between studies and might have been caused by differences in animal models, protein and mRNA measurement techniques, and follow-up time. However, the direction of up- or downregulations was concordant for most of the markers, allowing us to draw conclusion on the direction of the association of markers with anthracycline-induced cardiomyopathy. Second, animal models are different from anthracycline-induced cardiomyopathy seen in humans as higher anthracycline doses are used in animals (15 mg/kg in rodents corresponds to ±580 mg/m^2^ in humans), latency to cardiomyopathy development is longer in humans, and animal models lack exposure to aging-related cardiovascular risk factors. The conversion of animal follow-up times to human equivalent years, that allowed us to compare temporal changes in marker expression across species, should mainly be seen as indicative for the effects of anthracyclines in human perspective.

### Summary of temporal ECM remodeling patterns in anthracycline-induced cardiomyopathy

**Fig. 5:**
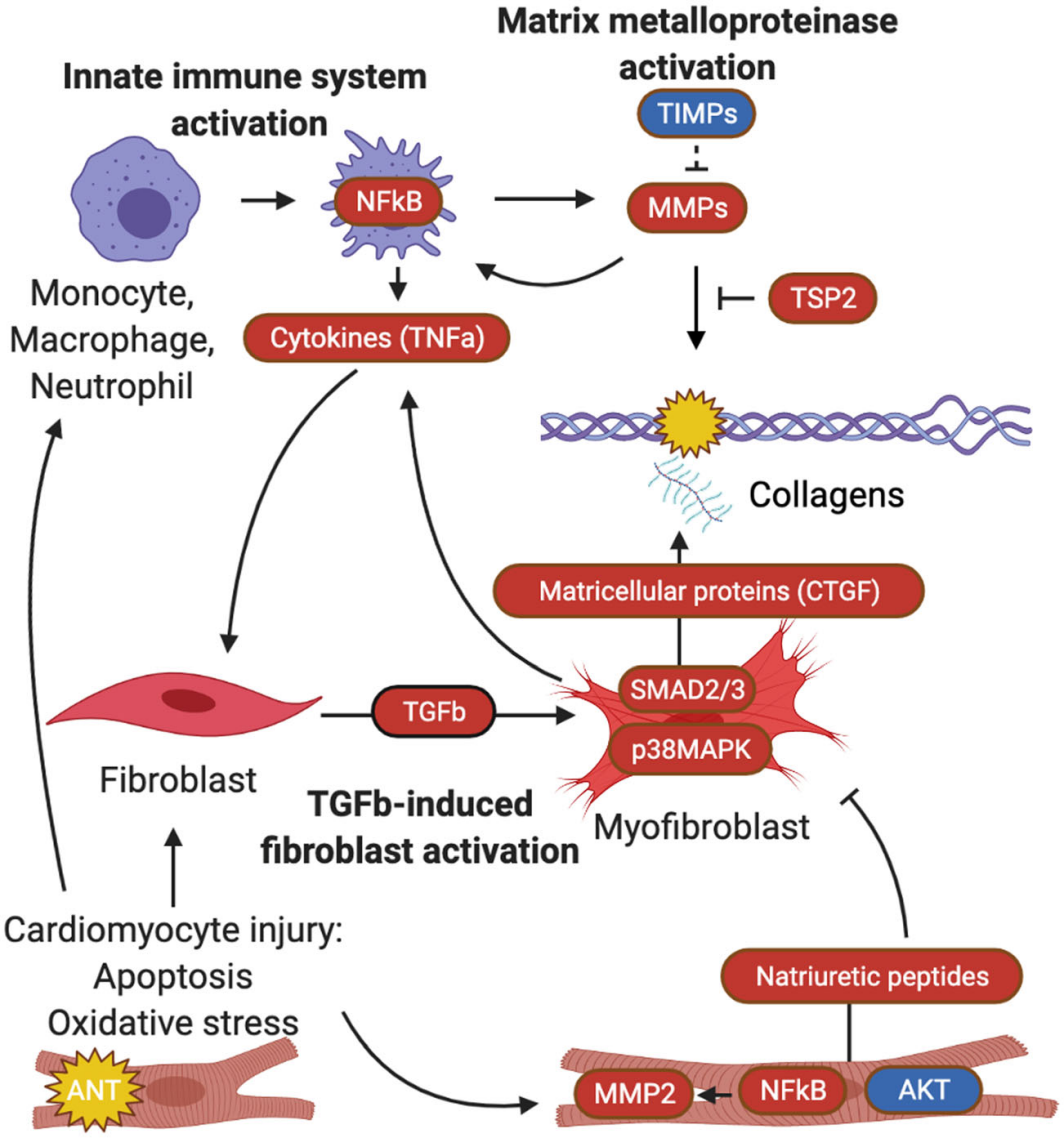
Summary of extracellular matrix remodeling in anthracycline-induced cardiomyopathy. Upregulated markers are in red; downregulated markers are in blue. AKT=phosphorylated protein kinase B, ANT=anthracyclines, CTGF=connective tissue growth factor, MMP=matrix metalloproteinase, NF-κB=nuclear factor-κB, p38 MAPK=p38 mitogen-activated protein kinases, SMAD=Small Mothers Against Decapentaplegic homolog, TGFb=transforming growth factor ß, TIMP=tissue inhibitor of metalloproteinase, TNF=tumor necrosis factor, TSP2=thrombospondin 2. Created with biorender.com

Based on our review and knowledge obtained from other cardiomyopathies [6, 7, 9, 53], we propose the following temporal ECM remodeling patterns in anthracycline-induced cardiomyopathy (Fig. 5). We distinguish three main processes that together contribute to the observed ECM remodeling. First, in response to anthracycline-induced cardiomyocyte injury (including oxidative stress and apoptosis) and release of DAMPS, the *innate immune system* is activated with release of pro-inflammatory cytokines by macrophages and other immune cells [9, 44]. The consistent increase in expression of NFkB we observed in our review reflects a strong innate immune response that is known to cause pathological ECM remodeling when persistently activated [48]. Similarly, we observed persistent upregulation of the pro-inflammatory cytokine TNFα, which also implicates a chronic pro-inflammatory state in anthracycline-induced cardiomyopathy.

Second, both oxidative stress and the subsequent inflammatory reaction can *activate MMPs* within cardiomyocytes, fibroblasts, and immune cells. ECM fragments released during matrix degradation by activated MMPs are itself pro-inflammatory and thus result in a positive feedback loop with chronic inflammation and matrix metalloproteinase activation [53]. In this review, we observed upregulation in MMPs (MMP2 and MMP9) with increasing levels of MMP9 in studies with longer follow-up, which is inadequately counteracted by TIMPs. This shows that this pathway is persistently activated in anthracycline-induced cardiomyopathy and that dysregulation might become more pronounced longer after anthracycline administration, possibly due to MMP9 secretion by invading immune cells.

Third, chronic inflammation as well as the release of DAMPs can trigger *TGFβ1 activation* and fibroblast to myofibroblast conversion and subsequent collagen synthesis [53]. We observed upregulation of markers in the TGFβ signaling pathway, such as CTGF that showed an early peak in expression and sustained upregulation in studies with longer follow-up after anthracyclines. Myofibroblasts are activated by chronic inflammation and they also secrete a number of pro-inflammatory cytokines themselves that are able to maintain the chronic inflammation that we observed in this review [54].

In summary, the innate immune system, MMPs, and the TGFβ signaling pathway are tightly connected to each other and are persistently activated in animals after exposure to anthracyclines without a clear diminish over time. Together the above processes are likely to contribute to the observed ECM remodeling with accumulation of interstitial fibrosis in the hearts of animals with anthracycline-induced cardiomyopathy.

## Supplementary Information

ESM 1 (DOCX 167 kb)

## Data Availability

Systematic review data is available in the manuscript and supplementary files.
