# Supplementary material for: Extracellular matrix remodeling in animal models of anthracycline-induced cardiomyopathy: a meta-analysis
Source: J Mol Med (Berl). 2021 May 29;99(9):1195–207. doi: 10.1007/s00109-021-02098-8 (PMC8367936; doi:10.1007/s00109-021-02098-8)
Supplement: Supplementary file 1 — (DOCX 167 kb) [file 109_2021_2098_MOESM1_ESM.docx]

**Supplemental material**

**SEARCH STRATEGY**

**Pubmed**

| **Anthracyclines:**  Antibiotics/Anti-bacterial agents[Mesh] OR Antibiotics/Anti-bacterial agents[tiab] OR Antineoplastic/toxicity[Mesh] OR Antineoplastic/toxicity[Tiab] OR Anthracyclines/adverse effects[Mesh] OR Anthracyclines/toxicity[Mesh] OR Anthracyclin[tiab] OR Antracyclin[tiab] OR Aclarubicin/toxicity[Mesh] OR Aclarubicin/toxicity[Tiab] OR Aclarubicin/adverse effects[Mesh] OR Aclarubicin/adverse effects[Tiab] OR Daunorubicin/toxicity[Mesh] OR Daunorubicin [tiab] OR Daunorubicin/adverse effects[Mesh] OR Daunorubucin/adverse effects[Tiab] OR Carubicin/toxicity[Mesh] OR Carubicin/toxicity[Tiab] OR Carubicin/adverse effects[Mesh] OR Carubicin/adverse effects[Tiab] OR Idarubicin/toxicity[Mesh] OR Idarubicin/toxicity[Tiab] OR Idarubicin/adverse effects[Mesh] OR Idarubicin/adverse effects[Tiab] OR Doxorubicin/toxicity[Mesh] OR Doxorubicin/toxicity[Tiab] OR Doxorubicin/adverse effects[Mesh] OR Doxorubicin[tiab] OR Nogalamycin/toxicity[Mesh] OR Nogalamycin/toxicity[Tiab] OR Nogalamycin/adverse effects[Mesh] OR Nogalamycin/adverse effects[Tiab] OR Plicamycin/toxicity[Mesh] OR Plicamycin/toxicity[Tiab] OR Plicamycin/adverse effects[Mesh] OR Plicamycin/adverse effects[Tiab] |
| --- |
| AND |
| **Cardiotoxicity:**  "Heart/adverse effects"[Mesh] OR "Heart/toxicity"[Mesh] OR "Ventricular Dysfunction"[Mesh] OR "Cardiotoxicity"[Mesh] OR cardiotoxicit*[tiab] OR ejection fraction[tiab] OR LVEF[tiab] OR "Stroke Volume"[Mesh] OR contractilit*[tiab] OR "Cardiomyopathies"[Mesh] OR cardiomyopath*[tiab] OR cardiac[tiab] OR myocard*[tiab] OR heart[tiab] OR ventricular[tiab] OR "Myocytes, Cardiac"[Mesh] OR "cardiomyocyt*"[tiab] OR "Heart"[Mesh] OR "Heart"[tiab] OR "Myocardium"[Mesh] OR "Myocardium"[tiab] OR "cardiac*"[tiab] OR "cardio*"[tiab] OR “ventric*”[tiab] |
| AND |
| **Extracellular matrix remodeling:**  "Matrix Metalloproteinases"[Mesh] OR "Matrix Metalloproteinase*"[tiab] OR "Extracellular Matrix"[Mesh] OR "Extracellular Matrix"[tiab] OR "Ventricular Remodeling/physiology"[mesh] OR “remodeling”[tiab] OR "collagen*"[tiab] OR "MMP*"[tiab] OR “fibros*”[tiab] OR “protein kinase B”[tiab] OR “p38”[tiab] OR “cystatin C”[tiab] OR “thrombospondin”[tiab] |

Search date: 5-12-2019, 511 hits.

**Embase**

| **Anthracyclines:**   1. (4-demethoxydaunorubicin or 4 demethoxydaunorubicin or 4-desmethoxydaunorubicin or 4 desmethoxydaunorubicin).mp. or exp idarubicin/ 2. (IMI 30 or IMI30 or IMI-30 or idarubicin hydrochloride).mp. 3. (NSC 256439 or NSC-256439 or NSC256349 or idarubicin or idarubic$).mp. 4. (4'-epiadriamycin or 4' epiadriamycin or 4'-epidoxorubicin or 4' epidoxorubicin or 4'-epi-doxorubicin or 4' epi doxorubicin).mp. 5. (4'-epi-adriamycin or 4' epi adriamycin or 4'-epi-DXR or 4' epi DXR).mp. 6. exp epirubicin/ or (epirubicin or epirubicin hydrochloride or epirubic$ or farmorubicin).mp. 7. (IMI-28 or IMI 28 or IMI28 or NSC 256942 or NSC-256942 or NSC256942).mp. 8. (adriablastine or adriblastin or adriablastin or adriamycin).mp. 9. (DOX-SL or DOX SL or doxorubicin hydrochloride or doxorubic$ or adramyc$).mp. 10. (dauno-rubidomycine or dauno rubidomycin or rubidomycin or rubomycin or daunomycin).mp. 11. (cerubidine or daunoblastin or daunoblastine or daunorubicin hydrochloride or daunorubic$).mp. 12. (NSC-82151 or NSC 82151 or NSC82151).mp. 13. (daunoxome or daunoxom$ or daunosom$ or doxil or caelyx or liposomal doxorubicin or myocet or doxorubicin or daunorubicin).mp. 14. exp DAUNORUBICIN DERIVATIVE/ or exp DAUNORUBICIN/ or exp IDARUBICIN DERIVATIVE/ or exp IDARUBICIN/ or exp DOXORUBICIN DERIVATIVE/ or exp DOXORUBICIN/ or exp EPIRUBICIN/ 15. (anthracyclin$ or anthracyclines).mp. or exp Anthracycline/ 16. anthracycline antibiotics.mp. or exp Anthracycline Antibiotic Agent/ 17. exp Anthracycline Derivative/ 18. 1 or 2 or 3 or 4 or 5 or 6 or 7 or 8 or 9 or 10 or 11 or 12 or 13 or 14 or 15 or 16 or 17 |
| --- |
| AND |
| **Cardiotoxicity:**   1. ventricular dysfunction.mp. or exp ventricular dysfunction/ 2. heart fail$.mp. 3. (cardiotox$ or cardi$ tox$).mp. 4. cardiac dysfunction$.mp. 5. cardiac function$.mp. 6. cardiac event$.mp. 7. cardiac fail$.mp. 8. echocardiograph$.mp. 9. exp heart ventricle function/ 10. exp cardiotoxicity/ 11. 19 or 20 or 21 or 22 or 23 or 24 or 25 or 26 or 27 or 28 |
| AND |
| **Extracellular matrix remodeling:**   1. Matrix metalloprotein$.mp 2. Extracellular matrix.mp 3. Remodel$.mp 4. Collagen$.mp 5. MMP$.mp 6. Fibros$.mp 7. 30 or 31 or 32 or 33 or 34 or 35 |
| 1. 18 AND 29 AND 36 2. limit 37 to animals |

Search date: 5-12-2019, 597 hits.

**SUPPLEMENTAL TABLES**

| **Table S1.** Overview of the included studies in the systematic review | | | | | | | | |
| --- | --- | --- | --- | --- | --- | --- | --- | --- |
| **Author** | **Animal model** | **Anthracycline derivative** | **Injection** | **Cumulative anthracycline dose (mg/kg)** | **Age at start of anthracycline treatment (weeks)** | **Weeks after first dose studied** | **Human equivalent years after first dose** | **Cardiomyopathy quantification (ACMP/control)** |
| Chan 2021 [1] | Mice, n=20 | DOX | IP | 24 | 8 | 4.0 | 1.7 | Histology  LVEF (46%/64%) |
| Chen 2015 [2] | Mice, n=20 | DOX | IP | 15.0 | 8 | 5.0 | 2.2 | histology  LVEF (47/60%) |
| Chen 2018 [3] | Mice, n=12 | DOX | IP | 15.0 | 8 | 6.0 | 2.6 | dP/dt+ (3428/6059 mmHg) |
| Johnson 2018 [4] | Mice, n=16 | DOX | IP | 12.0 | 8-12 | 8.0 | 3.4 | histology  LVEF (63/82%) |
| Ohlig 2018 [5] | Mice, n=24 | DOX | IP | 15.0 | 10-12 | 3.9 | 1.7 | LVEF (60/65%) |
| Rasanen 2016 [6] | Mice, n=14 | DOX | IP | 24.0 | 9-10 | 4.0 | 1.7 | histology  LVESV (31/18 uL), LVEDV (78/55 uL) |
| Russo 2019 [7] | Mice, n=20 | DOX | IP | 7.0 | 8-12 | 1.0 | 0.4 | histology  LVEF (75/84%) |
| Shi 2018 [8] | Mice, n=10 | DOX | IP | 24.0 | 8-9 | 3.0 | 1.3 | histology  FS (32/46%) |
| Singla 2015 [9] | Mice, n=12 | DOX | IP | 12.0 | NA | 5.0 | 2.2 | histology |
| Singla 2019 [10] | Mice, n=12 | DOX | IP | 12.0 | 8-12 | 3.0 | 1.3 | histology |
| Sun 2015 [11] | Mice, n=41 | DOX | IP | 18.0 | 7-8 | 6.0 | 2.6 | NA |
| Tocchetti 2014 [12] | Mice, n=16 | DOX | IP | 15.0 | 8-16 | 1.0 | 0.4 | histology  FS (49/60%) |
| van Almen 2011 [13] | Mice, n=13 | DOX | IP | 24.0 | 10-12 | 12.0 | 5.2 | histology  FS (35/36%) |
| Wang 2014 [14] | Mice, n=12 | DOX | IP | 32.0 | 8-12 | 5.0 | 2.2 | histology  LVEF (55/70%) |
| Wang 2017 [15] | Mice, n=22 | DOX | IP | 20.0 | 11 | 4.0 | 1.7 | histology  LVEF (66/77%) |
| Yi 2006 [16] | Mice, n=10 | DOX | IP | 3, 9, 18, 36 | 6-8 | 1, 3, 6, 12, 18 | 0.4, 1.3, 2.6, 5.2, 7.8 | FS at 18 weeks (46/73%) |
| Zhang 2012 [17] | Mice, n=14 | DOX | IP | 25.0 | 4 | 7.0 | 3 | histology  LVEF (42/54%) |
| Zhao 2010 [18] | Mice, n=16 | DOX | IP | 12.0 | 8-10 | 8.0 | 3.4 | histology  LVEF (29/57%) |
| Zhou 2015 [19] | Mice, n=8 | DOX | IV | 15.0 | 8 | 9.0 | 3.9 | FS (33/56%) |
| Gálan-Arriola 2021 [20] | Pigs, n=20 | DOX | IC | 2.25 | 12 | 16.0 | 1.7 | histology  LVEF (33/62%) |
| Goetzenich 2009 [21] | Pigs, n=12 | DOX | IC | 5-7 x 25mg (±2.9 mg/kg) | adults | 3.57-5 (studied when LVEF<50%) | 0.3 | histology  LVEF (38/76%) |
| Gyongyosi 2019 [22] | Pigs, n=11 | DOX | IV | 180 mg/m2 (±4.1 mg/kg) | 12 | 8.6 | 0.5 | LVEF (45/57%) |
| Adamcova 2010 [23] | Rabbits, n=19 | DNR | IV | 30.0 | adults | 10.0 | 1.9 | histology  dP/dt+ (5802/8688 mmHg) |
| Aupperle 2007 [24] | Rabbits, n=15 | DOX | IV | 18.0 | 12 | 8.0 | 1.5 | histology |
| Lencova 2014 [25] | Rabbits, n=16 | DNR | IV | 30.0 | NA | 20.0 | 3.8 | histology  FS (19/41%) |
| Potacova 2007 [26] | Rabbits, n=19 | DNR | IV | 30.0 | NA | 10.0 | 1.9 | histology |
| Rodrigues 2018 [27] | Rabbits, n=15 | DOX | IV | 16.0 | 16 | 9.0 | 1.7 | histology  LVEF (72/75%) |
| Sterba 2011 [28] | Rabbits, n=12 | DNR | IV | 30.0 | NA | 11.0 | 2.1 | histology  FS (28/42.5%) |
| Abu Gazia 2018 [29] | Rats, n=20 | DOX | IP | 17.5 | 12-16 | 3.0 | 1.2 | histology |
| Arafa 2014 [30] | Rats, n=20 | DOX | IP | 15.0 | adult | 2.0 | 0.8 | histology |
| Arozal 2010-05 [31] | Rats, n=11 | DNR | IV | 9.0 | 8 | 6.0 | 2.5 | histology  LVEF (60/79%) |
| Arozal 2010-08 [32] | Rats, n=15 | DNR | IP | 18.0 | 10-12 | 1.9 | 0.8 | histology  LVEF (49/83%) |
| Bartekova 2015 [33] | Rats, n=14 | DOX | IP | 15.0 | 11 | 11.0 | 4.5 | histology |
| Cappetta 2016 [34] | Rats, n=30 | DOX | IP | 15.0 | 12 | 2.0 | 0.8 | histology  LVEF (76/86%) |
| Cappetta 2017 [35] | Rats, n=35 | DOX | IP | 15.0 | 12 | 6.0 | 2.5 | LVEF (64/87%) |
| Chan 2011 [36] | Rats, n=26 | DOX | IV | 15.0 | adults | 11.0 | 4.5 | histology  FS (34/45%) |
| Chen 2014 [37] | Rats, n=16 | DOX | IP | 15.0 | 10-11 | 4.0 | 1.6 | histology |
| Chen 2016 [38] | Rats, n=12 | DOX | IP | 17.5 | 4 | 3.0 | 1.2 | histology |
| Chua 2016 [39] | Rats, n=16 | DOX | IP | 28.0 | adults | 11.4 | 4.7 | histology  LVEF (49/74%) |
| Das 2011 [40] | Rats, n=12 | DOX | IP | 9.0 | 6 | 4.0 | 1.6 | histology |
| El-Said 2019 [41] | Rats, n=16 | DOX | IP | 15.0 | 8-10 | 2.3 | 0.9 | histology |
| Gordiienko 2014 [42] | Rats, n=16 | DOX | IP | 4.0 | adults | 4.0 | 1.6 | histology |
| Hang 2017 [43] | Rats, n=10 | DOX | IP | 15.0 | adults | 2.0 | 0.8 | histology  LVEF (62/91%) |
| Hong 2017 [44] | Rats, n=12 | DOX | IP | 15.0 | 8-12 | 3.0 | 1.2 | histology  LVEF (54/65%) |
| Ivanova 2012 [45] | Rats, n=32 | DOX | IP | 15.0 | 10 | 7, 11 | 2.9, 4.5 | histology |
| Levick 2018 [46] | Rats, n=12 | DOX | IP | 9.0 | 8 | 1.0 | 0.4 | LVEF (70/80%) |
| Lim 2013 [47] | Rats, n=20 | DOX | IP | 15.0 | 9 | 0.4, 0.7, 1, 2 | 0.2, 0.3, 0.4, 0.8, | histology |
| Liu 2016 [48] | Rats, n=12 | DOX | IP | 10.0 | adults | 2.0 | 0.8 | histology  LVEF (40/71%) |
| Lou 2005 [49] | Rats, n=12 | DOX | IP | 15.0 | adults | 5.0 | 2.1 | dP/dt+ (8631/10931 mmHg) |
| Mantawy 2017 [50] | Rats, n=20 | DOX | IP | 20.0 | NA | 0.3 | 0.1 | histology |
| Medeiros 2019 [51] | Rats, n=16 | DOX | IP | 10.0 | 12 | 2.4, 3.4, 5.4 | 1.0, 1.4, 2.23 | histology  5.4 weeks dP/dt+ (851/1903 mmHg) |
| Mohamed 2018 [52] | Rats, n=20 | DOX | IP | 15.0 | adult | 2.0 | 0.8 | histology |
| Pandey 2019 [53] | Rats, n=10 | DOX | IP | 30.0 | 10 | 7.0 | 2.9 | histology  LVEF (35/44%) |
| Richard 2011 [54] | Rats, n=16 | DOX | IP | 10.0 | NA | 10.0 | 4.1 | histology  dP/dt+ (2827/3807 mmHg) |
| Shaker 2010 [55] | Rats, n=20 | DOX | IP | 15.0 | NA | 9.0 | 3.7 | histology |
| Shati 2019 [56] | Rats, n=20 | DOX | IP | 15.0 | 7 | 5.0 | 2.1 | histology  dP/dt+ (1342/4122 mmHg) |
| Sun 2017 [57] | Rats, n=17 | DOX | IP | 15.0 | NA | 6.0 | 2.5 | histology  LVEF (53/83%) |
| Tian 2017 [58] | Rats, n=20 | DOX | IP | 18.0 | 6-7 | 7.0 | 2.9 | histology  LVEF (57/84%) |
| Vacchi-Suzzi 2012 [59] | Rats, n=12 | DOX | IV | 6-18 | 11 | 2, 4 ,6 | 0.8, 1.6, 2.5 | histology |
| Wergeland 2011 [60] | Rats, n=14 | DNR | IP | 12.0 | NA | 2.0 | 0.8 | NA |
| Wu 2016 [61] | Rats, n=16 | DOX | IP | 12.0 | NA | 6.0 | 2.5 | histology |
| Xiang 2009 [62] | Rats, n=24 | DOX | IV | 15.0 | NA | 11.0 | 4.5 | histology  FS (32/44%) |
| Xiao 2012 [63] | Rats, n=30 | DOX | IP | 9.0 | 8 | 4.9 | 2 | histology |
| Yu 2013-06 [64] | Rats, n=20 | DOX | IP | 17.5 | 0 | 2.0 | 0.8 | histology  LVEF (48/72%) |
| Yu 2013-12 [65] | Rats, n=30 | DOX | IV | 40.0 | 2 | 8.0 | 3.3 | histology  LVEF (65/72%) |
| Yu 2014 [66] | Rats, n=18 | DOX | IP | 15.0 | 8 | 20.0 | 8.2 | histology  LVEF (66/88%) |
| Zhang 2017 [67] | Rats, n=40 | DOX | IP | 33.6 | adults | 8.0 | 3.3 | histology  LVEF (64/84%) |
| Zhang 2018 [68] | Rats, n=20 | DOX | IP | 14.0 | adults | 7.0 | 2.9 | histology |
| Abbreviations: DOX=doxorubicin, DNR=daunorubicin, IC=intracoronary, IP=intraperitoneally, IV=intravenously, LVEF=left ventricular ejection fraction. | | | | | | | | |

| **Table S2.** Histologic observations in animals with anthracycline-induced cardiomyopathy in the included studies. | |
| --- | --- |
| **Author** | **Histologic observations** |
| Abu Gazia 2018 | Cardiomyocyte disorganization, sarcoplasmic eosinophilia, pyknotic nuclei, interstitial mononuclear infiltration, congestion, interstitial fibrosis |
| Adamcova 2010 | Interstitial and perivascular fibrosis |
| Arafa 2014 | Necrosis, rupture of cardiac muscle fibers, myocyte damage, interfibrillar congestion, interstitial fibrosis |
| Arozal 2010-05 | Interstitial fibrosis, perinuclear vacuolization, myocardial degeneration, interstitial edema |
| Arozal 2010-08 | Edema, hemorrhage and congestion |
| Aupperle 2007 | Interstitial fibrosis |
| Bartekova 2015 | Increase in density of extracellular matrix proteins, collagen, vacuolization of cardiomyocytes and fibroblasts |
| Cappetta 2016 | Enhanced fibroblast to myofibroblast transformation, collagen deposition |
| Cappetta 2017 | NA |
| Chan 2011 | Decrease in myofibrils, increase in cytoplasmic vacuolization and cardiomyopathy score |
| Chan 2021 | Increased nuclear size, crowding of cardiomyocytes, cardiomyocyte paucity or dropout with increased  interstitial oedema, myofibrillar disorganization, interstitial fibrosis |
| Chen 2014 | Atrophic/hypertrophic cardiomyocytes, interstitial fibrosis |
| Chen 2015-12 | Increase in interstitial fibrosis area |
| Chen 2016 | Interstitial fibrosis, cardiomyocyte disarrangement |
| Chen 2018 | NA |
| Chua 2016 | Increase in interstitial fibrosis area |
| Das 2011 | Dose responsive reduction in cardiomyocyte viability and decrease in mitochondrial membrane potential |
| El-Said 2019 | Focal necrosis and hemorrhage |
| Gálan-Arriola 2021 | Increase in interstitial fibrosis area |
| Goetzenich 2009 | Multifocal necrosis, increase in fibroblasts, leucocyte infiltration, increased collagen I/III ratio |
| Gordiienko 2014 | Interstitial fibrosis and hemorrhages |
| Gyongyosi 2019 | NA |
| Hang 2017 | Disorganized myofibers and swollen mitochondria, disordered myocardial fibers, increase in collagen volume fraction |
| Hong 2017 | Increase in collagen content |
| Ivanova 2012 | Reduction in myofibrils, increased cytoplasmic vacuolization of cardiomyocytes, disorganization of extracellular matrix with increased density of extracellular matrix proteins, damaged endothelial cells of the capillaries. |
| Johnson 2018 | Cardiomyocyte hypertrophy, perivascular fibrosis |
| Lencova-Popelova 2014 | Myofibrillar loss, cardiomyocyte vacuolization, cardiomyocyte enlargement, focal replacement fibrosis |
| Levick 2018 | NA |
| Lim 2013 | Cytoplasmic vacuolization, loss of myofibrils, and nuclear degeneration |
| Liu 2016 | NA |
| Lou 2005 | NA |
| Mantawy 2017 | Myofibrillar loss, cytoplasmic vacuolization, inflammatory cell infiltration, edema, congestion and nuclear pyknosis |
| Medeiros-Lima 2019 | Swollen and vacuolated cardiomyocytes, myofilament disarray, mitochondrial damage and interstitial fibrosis |
| Mohamed 2018 | Fragmentation of cardiac muscle fibers, pyknotic nuclei, mononuclear cell infiltration between cardiomyocytes, cytoplasmic vacuolization |
| Ohlig 2018 | NA |
| Pandey 2019 | Interstitial fibrosis, cardiomyocyte disarrangement |
| Potacova 2007 | Interstitial fibrosis, cardiomyocyte disarrangement and vacuolization |
| Rasanen 2016 | Damaged endothelial cells and atrophied mitochondria |
| Richard 2011 | Collagen I and III deposition mainly in areas around coronary vessels |
| Rodrigues 2018 | Shift in titin isoform from the stiff N2B to the more compliant N2BA, cardiomyocyte hypertrophy, interstitial fibrosis |
| Russo 2019 | Cardiomyocyte hypertrophy, interstitial fibrosis |
| Shaker 2010 | Cardiomyocyte fragmentation, separation of myofibrils, extravasation of blood |
| Shati 2019 | Increase in collagen fibers, degenerated myofibrils, damaged mitochondria |
| Shi 2018 | Autophagic vacuoles, collagen deposition |
| Singla 2015 | Interstitial fibrosis |
| Singla 2019 | Cytoplasmic vacuolization, cardiomyocyte hypertrophy, myofibril loss, interstitial and perivascular fibrosis |
| Sterba 2011 | Gradually degenerating and enlarged cardiomyocytes with vacuolated cytoplasm and loss of myofibrils. Dead cardiomyocytes are subsequently replaced by connective tissue with a gradually increasing amount of collagen fibers |
| Sun 2017 | NA |
| Sun 2015 | NA |
| Tian 2017 | Interstitial fibrosis |
| Tocchetti 2014 | Interstitial fibrosis |
| Vacchi-Suzzi 2012 | Cardiomyocyte vacuolization |
| van Almen 2011 | Interstitial fibrosis, erythrocytes |
| Wang 2014 | Interstitial fibrosis, myocyte hypertrophy |
| Wang 2017 | Interstitial fibrosis |
| Wergeland 2011 | NA |
| Wu 2016 | Thick and disorganized muscle fibers, vacuolization, inflammatory cell infiltration, interstitial fibrosis |
| Xiang 2009 | Myofibrillar loss, cytoplasmic vacuolization, swelling and vacuolization of mitochondria, damaged sarcotubular system |
| Xiao 2012 | Cytoplasmic vacuolization, myofibrillar loss, mitochondrial edema, chromatin condensation, cardiomyocyte necrosis. |
| Yi 2006 | Cardiomyocyte vacuole formation and heterogeneous cell size |
| Yu 2013-06 | Cardiomyocyte necrosis |
| Yu 2013-12 | Interstitial fibrosis, disorganized collagenous fibers, thicker type I collagenous fibers compared to controls |
| Yu 2014 | NA |
| Zhang 2012 | Patchy fibrosis, cardiomyocyte vacuolization |
| Zhang 2017 | Cardiomyocyte disarray, partial necrosis, very few fibroblasts, minimal inflammatory cells around the necrotic myocardium, interstitial and perivascular fibrosis |
| Zhang 2018 | Myocardial fiber degeneration |
| Zhao 2010 | Cardiomyocyte cross sectional area decrease and interstitial fibrosis |
| Zhou 2015 | NA |
| Abbreviations: NA=not available. | |

| **Table S3.** Meta-regression results of studies in mice, rats and rabbits. Proteins and mRNAs that were significant in meta-analysis (p<0.05 and ROM>1.2 or <0.83) are shown and their association with time after first anthracycline injection, left ventricular systolic function and cardiomyocyte apoptosis. | | | | | | | | | | | |
| --- | --- | --- | --- | --- | --- | --- | --- | --- | --- | --- | --- |
| **Pathway** | **Protein/mRNA (mR)** | **Studies (n)** | **HE years after anthracyclines, median (range)** | **Time**  **ß** | **Time**  **p** | **LVEF**  **ß** | **LVEF**  **p** | **Fibrosis**  **ß** | **Fibrosis**  **p** | **TUNEL**  **ß** | **TUNEL**  **p** |
| Collagen synthesis | CollagenXV_mR | 1 | 7.8 (7.8-7.8) | NA | NA | NA | NA | NA | NA | NA | NA |
|  | CollagenIV | 1 | 2.9 (2.9-2.9) | NA | NA | NA | NA | NA | NA | NA | NA |
|  | CollagenIV_mR | 1 | 3.8 (3.8-3.8) | NA | NA | NA | NA | NA | NA | NA | NA |
|  | CollagenIII_mR | 4 | 3.1 (2.1-8.2) | NA | NA | NA | NA | NA | NA | NA | NA |
|  | CollagenI | 4 | 2.3 (0.8-8.2) | NA | NA | NA | NA | NA | NA | NA | NA |
|  | CollagenI_mR | 7 | 2.5 (0.8-8.2) | -0.05 | 0.55 | 0.03 | 0.39 | -0.03 | 0.35 | NA | NA |
| Matrix metalloproteinases | proMMP9 | 1 | 1.6 (1.6-1.6) | NA | NA | NA | NA | NA | NA | NA | NA |
|  | proMMP2 | 1 | 1.6 (1.6-1.6) | NA | NA | NA | NA | NA | NA | NA | NA |
|  | **MMP9** | 13 | 2.2 (0.2-5.2) | **0.22** | **0.04** | -0.01 | 0.93 | 0.02 | 0.25 | -0.08 | 0.37 |
|  | **MMP2_mR** | 9 | 1.7 (0.4-5.2) | -0.50 | 0.11 | 0.06 | 0.92 | 0.30 | 0.10 | **0.33** | **0.02** |
|  | NTT_MMP2_mR | 1 | 1.7 (1.7-1.7) | NA | NA | NA | NA | NA | NA | NA | NA |
|  | **MMP2** | 19 | 1.8 (0.2-5.2) | 0.01 | 0.86 | **-0.11** | **0.02** | 0.03 | 0.24 | 0.08 | 0.73 |
|  | TSP2 | 1 | 5.2 (5.2-5.2) | NA | NA | NA | NA | NA | NA | NA | NA |
| TGFb family signaling | BMP | 2 | 3.4 (2.2-4.7) | NA | NA | NA | NA | NA | NA | NA | NA |
|  | SMAD3 | 4 | 2.3 (0.8-4.7) | NA | NA | NA | NA | NA | NA | NA | NA |
|  | **TGFb1** | 13 | 2.1 (0.8-4.7) | -0.19 | 0.27 | -0.07 | 0.30 | 0.01 | 0.69 | **0.19** | **0.03** |
|  | **CTGF** | 6 | 1 (0.4-1.7) | **-1.70** | **0.02** | 0.31 | 0.47 | -0.02 | 0.82 | 0.23 | 0.18 |
|  | vimentin_mR | 1 | 7.8 (7.8-7.8) | NA | NA | NA | NA | NA | NA | NA | NA |
|  | vimentin | 2 | 3 (2.1-3.8) | NA | NA | NA | NA | NA | NA | NA | NA |
|  | BDNF | 1 | 0.8 (0.8-0.8) | NA | NA | NA | NA | NA | NA | NA | NA |
|  | pJAK2 | 1 | 2.1 (2.1-2.1) | NA | NA | NA | NA | NA | NA | NA | NA |
|  | p38MAPK | 9 | 1.6 (0.8-3.9) | -0.09 | 0.54 | -0.04 | 0.06 | NA | NA | NA | NA |
| AKT signaling | mTOR_mR | 1 | 3.3 (3.3-3.3) | NA | NA | NA | NA | NA | NA | NA | NA |
|  | pAKT | 17 | 2.9 (0.1-5.2) | 0.05 | 0.63 | -0.01 | 0.84 | -0.02 | 0.19 | -0.01 | 0.77 |
| Immune system | NLRP3 | 1 | 1.3 (1.3-1.3) | NA | NA | NA | NA | NA | NA | NA | NA |
|  | TLR4 | 1 | 1.3 (1.3-1.3) | NA | NA | NA | NA | NA | NA | NA | NA |
|  | GAL3 | 1 | 2.5 (2.5-2.5) | NA | NA | NA | NA | NA | NA | NA | NA |
|  | GAL3_mR | 1 | 2.5 (2.5-2.5) | NA | NA | NA | NA | NA | NA | NA | NA |
|  | IL10 | 1 | 1.3 (1.3-1.3) | NA | NA | NA | NA | NA | NA | NA | NA |
|  | CD206 macrophages | 2 | 2.4 (1.3-3.4) | NA | NA | NA | NA | NA | NA | NA | NA |
|  | IL18 | 1 | 1.3 (1.3-1.3) | NA | NA | NA | NA | NA | NA | NA | NA |
|  | IL1b | 3 | 1.7 (1.3-4.7) | NA | NA | NA | NA | NA | NA | NA | NA |
|  | IL6 | 5 | 1.7 (1.2-2.9) | 0.26 | 0.63 | 0.05 | 0.80 | -0.03 | 0.81 | NA | NA |
|  | TNFa | 6 | 2.3 (0.8-4.7) | -0.01 | 0.86 | -0.02 | 0.82 | 0.01 | 0.83 | NA | NA |
|  | LMO4_mR | 1 | 7.8 (7.8-7.8) | NA | NA | NA | NA | NA | NA | NA | NA |
|  | pFAK | 1 | 1.3 (1.3-1.3) | NA | NA | NA | NA | NA | NA | NA | NA |
|  | NFkB | 7 | 1.6 (0.1-4.7) | 0.04 | 0.77 | -0.05 | 0.21 | 0.02 | 0.41 | -0.05 | 0.16 |
| Cardiac hypertrophy | GATA4_mR | 1 | 3.8 (3.8-3.8) | NA | NA | NA | NA | NA | NA | NA | NA |
|  | **ANP** | 5 | 1.7 (0.4-4.1) | -0.41 | 0.14 | -0.03 | 0.56 | **0.76** | **0.01** | -0.11 | 0.52 |
|  | **BNP** | 8 | 1.7 (0.8-4.7) | **0.32** | **0.03** | **-0.10** | **0.04** | 0.03 | 0.291 | -0.06 | 0.65 |
|  | CARP_mR | 1 | 3.8 (3.8-3.8) | NA | NA | NA | NA | NA | NA | NA | NA |
|  | CHRF | 1 | 2.6 (2.6-2.6) | NA | NA | NA | NA | NA | NA | NA | NA |
| Abbreviations: ß=coefficient from meta-regression, HE=human equivalent, LVEF=left ventricular ejection fraction, mR=messenger RNA, NTT=N-terminal truncated, p=p value from meta-regression, TUNEL=terminal deoxynucleotidyl transferase dUTP nick end labelling. | | | | | | | | | | | |

| **Table S4.** SYRCLE risk of bias assessment. | | | | | | | | | | | |
| --- | --- | --- | --- | --- | --- | --- | --- | --- | --- | --- | --- |
| **Author** | **Animal** | **Selection bias** | | | **Performance bias** | | **Detection bias** | | **Attrition bias** | **Reporting bias** | **Other** |
|  |  | Sequence generation | Similar baseline characteristics | Allocation concealment | Random housing | Blinding of caregivers/investigators | Random outcome assessment | Blinding outcome assessor | No incomplete outcome data or properly adressed | Free from selective outcome reporting | Other potential biases? |
| Abu Gazia 2018 | rats | yes | yes | yes | unk | unk | unk | unk | unk | unk | - |
| Adamcova 2010 | rabbits | unk | unk | unk | unk | unk | unk | unk | unk | unk | - |
| Arafa 2014 | rats | unk | unk | unk | unk | unk | unk | unk | no | unk | - |
| Arozal 2010-05 | rats | yes | yes | yes | unk | unk | unk | unk | no | unk | - |
| Arozal 2010-08 | rats | yes | yes | yes | unk | unk | unk | unk | no | unk | - |
| Aupperle 2007 | rabbits | yes | yes | yes | unk | unk | unk | unk | unk | unk | - |
| Bartekova 2015 | rats | unk | unk | unk | unk | unk | unk | unk | unk | unk | - |
| Cappetta 2016 | rats | yes | yes | yes | unk | unk | unk | unk | yes | unk | - |
| Cappetta 2017 | rats | yes | unk | unk | unk | unk | unk | unk | no | unk | - |
| Chan 2011 | rats | yes | yes | yes | unk | unk | unk | unk | yes | unk | - |
| Chan 2021 | mice | yes | yes | yes | unk | yes | yes | yes | yes | unk | - |
| Chen 2014 | rats | unk | unk | unk | unk | unk | unk | unk | unk | unk | - |
| Chen 2015 | mice | yes | yes | yes | unk | unk | unk | unk | no | unk | - |
| Chen 2016 | rats | yes | yes | yes | unk | unk | unk | unk | yes | unk | - |
| Chen 2018 | mice | yes | yes | yes | unk | unk | unk | unk | yes | unk | - |
| Chua 2016 | rats | yes | yes | yes | unk | yes | yes | yes | yes | unk | - |
| Das 2011 | rats | yes | yes | yes | unk | unk | unk | unk | yes | unk | - |
| El-Said 2019 | rats | yes | yes | yes | unk | unk | unk | unk | yes | unk | - |
| Gálan-Arriola 2021 | pigs | yes | yes | yes | unk | yes | yes | yes | yes | unk | - |
| Goetzenich 2009 | pigs | NA | NA | NA | NA | NA | NA | NA | yes | unk | no controls |
| Gordiienko 2014 | rats | unk | unk | unk | unk | unk | unk | unk | unk | unk | - |
| Gyongyosi 2019 | pigs | yes | yes | yes | unk | yes | yes | yes | yes | unk | - |
| Hang 2017 | rats | unk | unk | unk | unk | unk | unk | unk | yes | unk | - |
| Hong 2017 | rats | unk | unk | unk | unk | unk | unk | unk | unk | unk | - |
| Ivanova 2012 | rats | unk | unk | unk | unk | unk | unk | unk | unk | unk | - |
| Johnson 2018 | mice | unk | unk | unk | unk | unk | unk | unk | yes | unk | - |
| Lencova 2014 | rabbits | yes | yes | yes | unk | unk | unk | unk | yes | unk | - |
| Levick 2018 | rats | yes | yes | yes | unk | unk | unk | unk | yes | unk | - |
| Lim 2013 | rats | yes | yes | yes | unk | unk | unk | unk | unk | unk | - |
| Liu 2016 | rats | yes | yes | yes | unk | unk | unk | unk | yes | unk | - |
| Lou 2005 | rats | unk | unk | unk | unk | unk | unk | unk | no | unk | - |
| Mantawy 2017 | rats | yes | yes | yes | unk | unk | unk | unk | yes | unk | - |
| Medeiros 2019 | rats | unk | unk | unk | unk | unk | unk | unk | unk | unk | - |
| Mohamed 2018 | rats | unk | unk | unk | unk | unk | unk | unk | unk | unk | - |
| Ohlig 2018 | mice | yes | yes | yes | unk | unk | unk | unk | no | unk | - |
| Pandey 2019 | rats | yes | yes | yes | unk | unk | unk | unk | no | unk | - |
| Potacova 2007 | rabbits | unk | unk | unk | unk | unk | unk | unk | unk | unk | - |
| Rasanen 2016 | mice | unk | unk | unk | unk | unk | unk | unk | unk | unk | - |
| Richard 2011 | rats | unk | unk | unk | unk | unk | unk | unk | no | unk | - |
| Rodrigues 2018 | rabbits | unk | unk | unk | unk | unk | yes | yes | no | unk | - |
| Russo 2019 | mice | yes | yes | yes | unk | yes | yes | yes | unk | unk | - |
| Shaker 2010 | rats | unk | unk | unk | unk | unk | unk | unk | unk | unk | - |
| Shati 2019 | rats | yes | yes | yes | unk | yes | yes | unk | yes | unk | - |
| Shi 2018 | mice | unk | unk | unk | unk | unk | unk | unk | unk | unk | - |
| Singla 2015 | mice | unk | unk | unk | unk | unk | unk | yes | no | unk | - |
| Singla 2019 | mice | unk | unk | unk | unk | unk | unk | unk | no | unk | - |
| Sterba 2011 | rabbits | unk | unk | unk | unk | unk | unk | unk | unk | unk | - |
| Sun 2015 | mice | unk | unk | unk | unk | unk | unk | unk | unk | unk | - |
| Sun 2017 | rats | yes | yes | yes | unk | unk | unk | unk | no | unk | - |
| Tian 2017 | rats | yes | yes | yes | unk | unk | unk | unk | unk | unk | - |
| Tocchetti 2014 | mice | unk | unk | unk | unk | yes | yes | yes | unk | unk | - |
| Vacchi 2012 | rats | unk | unk | unk | unk | unk | unk | unk | unk | unk | - |
| van Almen 2011 | mice | unk | unk | unk | unk | unk | unk | unk | yes | unk | - |
| Wang 2014 | mice | yes | yes | yes | unk | yes | unk | yes | yes | unk | - |
| Wang 2017 | mice | unk | unk | unk | unk | unk | unk | unk | yes | unk | - |
| Wergeland 2011 | rats | unk | unk | unk | unk | unk | unk | unk | no | unk | - |
| Wu 2016 | rats | yes | yes | yes | unk | yes | yes | yes | yes | unk | - |
| Xiang 2009 | rats | yes | yes | yes | unk | yes | yes | yes | unk | unk | - |
| Xiao 2012 | rats | yes | yes | yes | unk | unk | unk | unk | yes | unk | - |
| Yi 2006 | mice | unk | unk | unk | unk | yes | yes | yes | yes | unk | - |
| Yu 2013-06 | rats | unk | unk | unk | unk | yes | yes | yes | yes | unk | - |
| Yu 2013-12 | rats | unk | unk | unk | unk | unk | unk | unk | yes | unk | - |
| Yu 2014 | rats | yes | yes | yes | unk | yes | yes | yes | yes | unk | - |
| Zhang 2012 | mice | unk | unk | unk | unk | unk | unk | unk | no | unk | - |
| Zhang 2017 | rats | yes | yes | yes | unk | unk | unk | unk | unk | unk | - |
| Zhang 2018 | rats | yes | yes | yes | unk | unk | unk | unk | unk | unk | - |
| Zhao 2010 | mice | unk | unk | unk | unk | unk | unk | yes | no | unk | - |
| Zhou 2015 | mice | unk | unk | unk | unk | unk | unk | unk | no | unk | - |

**REFERENCES**

1. Chan BYH, Roczkowsky A, Cho WJ, Poirier M, Sergi C, Keschrumrus V, Churko JM, Granzier H, Schulz R (2021) MMP inhibitors attenuate doxorubicin cardiotoxicity by preventing intracellular and extracellular matrix remodelling. Cardiovascular research 117 (1):188-200. doi:10.1093/cvr/cvaa017

2. Chen YL, Chung SY, Chai HT, Chen CH, Liu CF, Chen YL, Huang TH, Zhen YY, Sung PH, Sun CK, Chua S, Lu HI, Lee FY, Sheu JJ, Yip HK (2015) Early Administration of Carvedilol Protected against Doxorubicin-Induced Cardiomyopathy. The Journal of pharmacology and experimental therapeutics 355 (3):516-527. doi:10.1124/jpet.115.225375

3. Chen L, Yan KP, Liu XC, Wang W, Li C, Li M, Qiu CG (2018) Valsartan regulates TGF-beta/Smads and TGF-beta/p38 pathways through lncRNA CHRF to improve doxorubicin-induced heart failure. Archives of pharmacal research 41 (1):101-109. doi:10.1007/s12272-017-0980-4

4. Johnson TA, Singla DK (2018) PTEN inhibitor VO-OHpic attenuates inflammatory M1 macrophages and cardiac remodeling in doxorubicin-induced cardiomyopathy. American journal of physiology Heart and circulatory physiology 315 (5):H1236-h1249. doi:10.1152/ajpheart.00121.2018

5. Ohlig J, Henninger C, Zander S, Merx M, Kelm M, Fritz G (2018) Rac1-mediated cardiac damage causes diastolic dysfunction in a mouse model of subacute doxorubicin-induced cardiotoxicity. Archives of toxicology 92 (1):441-453. doi:10.1007/s00204-017-2017-7

6. Rasanen M, Degerman J, Nissinen TA, Miinalainen I, Kerkela R, Siltanen A, Backman JT, Mervaala E, Hulmi JJ, Kivela R, Alitalo K (2016) VEGF-B gene therapy inhibits doxorubicin-induced cardiotoxicity by endothelial protection. Proceedings of the National Academy of Sciences of the United States of America 113 (46):13144-13149. doi:10.1073/pnas.1616168113

7. Russo M, Guida F, Paparo L, Trinchese G, Aitoro R, Avagliano C, Fiordelisi A, Napolitano F, Mercurio V, Sala V, Li M, Sorriento D, Ciccarelli M, Ghigo A, Hirsch E, Bianco R, Iaccarino G, Abete P, Bonaduce D, Calignano A, Berni Canani R, Tocchetti CG (2019) The novel butyrate derivative phenylalanine-butyramide protects from doxorubicin-induced cardiotoxicity. European journal of heart failure 21 (4):519-528. doi:10.1002/ejhf.1439

8. Shi J, Surma M, Wei L (2018) Disruption of ROCK1 gene restores autophagic flux and mitigates doxorubicin-induced cardiotoxicity. Oncotarget 9 (16):12995-13008. doi:10.18632/oncotarget.24457

9. Singla DK (2015) Akt-mTOR Pathway Inhibits Apoptosis and Fibrosis in Doxorubicin-Induced Cardiotoxicity Following Embryonic Stem Cell Transplantation. Cell transplantation 24 (6):1031-1042. doi:10.3727/096368914x679200

10. Singla DK, Johnson TA, Dargani ZT (2019) Exosome treatment enhances anti-inflammatory M2 macrophages and reduces inflammation-induced pyroptosis in doxorubicin-induced cardiomyopathy. Cells 8 (10):1224. doi:<http://dx.doi.org/10.3390/cells8101224>

11. Sun Z, Schriewer J, Tang M, Marlin J, Taylor F, Shohet RV, Konorev EA (2016) The TGF-beta pathway mediates doxorubicin effects on cardiac endothelial cells. Journal of molecular and cellular cardiology 90:129-138. doi:10.1016/j.yjmcc.2015.12.010

12. Tocchetti CG, Carpi A, Coppola C, Quintavalle C, Rea D, Campesan M, Arcari A, Piscopo G, Cipresso C, Monti MG, De Lorenzo C, Arra C, Condorelli G, Di Lisa F, Maurea N (2014) Ranolazine protects from doxorubicin-induced oxidative stress and cardiac dysfunction. European journal of heart failure 16 (4):358-366. doi:10.1002/ejhf.50

13. van Almen GC, Swinnen M, Carai P, Verhesen W, Cleutjens JP, D'Hooge J, Verheyen FK, Pinto YM, Schroen B, Carmeliet P, Heymans S (2011) Absence of thrombospondin-2 increases cardiomyocyte damage and matrix disruption in doxorubicin-induced cardiomyopathy. Journal of molecular and cellular cardiology 51 (3):318-328. doi:10.1016/j.yjmcc.2011.05.010

14. Wang X, Wang XL, Chen HL, Wu D, Chen JX, Wang XX, Li RL, He JH, Mo L, Cen X, Wei YQ, Jiang W (2014) Ghrelin inhibits doxorubicin cardiotoxicity by inhibiting excessive autophagy through AMPK and p38-MAPK. Biochem Pharmacol 88 (3):334-350. doi:10.1016/j.bcp.2014.01.040

15. Wang S, Wang Y, Zhang Z, Liu Q, Gu J (2017) Cardioprotective effects of fibroblast growth factor 21 against doxorubicin-induced toxicity via the SIRT1/LKB1/AMPK pathway. Cell death & disease 8 (8):e3018. doi:10.1038/cddis.2017.410

16. Yi X, Bekeredjian R, DeFilippis NJ, Siddiquee Z, Fernandez E, Shohet RV (2006) Transcriptional analysis of doxorubicin-induced cardiotoxicity. American journal of physiology Heart and circulatory physiology 290 (3):H1098-1102. doi:10.1152/ajpheart.00832.2005

17. Zhang S, Liu X, Bawa-Khalfe T, Lu L-S, Lyu YL, Liu LF, Yeh ETH (2012) Identification of the molecular basis of doxorubicin-induced cardiotoxicity. Nature medicine 18:1639. doi:10.1038/nm.2919

18. Zhao Y, McLaughlin D, Robinson E, Harvey AP, Hookham MB, Shah AM, McDermott BJ, Grieve DJ (2010) Nox2 NADPH oxidase promotes pathologic cardiac remodeling associated with Doxorubicin chemotherapy. Cancer Res 70 (22):9287-9297. doi:10.1158/0008-5472.Can-10-2664

19. Zhou L, Chen L, Wang J, Deng Y (2015) Astragalus polysaccharide improves cardiac function in doxorubicin-induced cardiomyopathy through ROS-p38 signaling. International journal of clinical and experimental medicine 8 (11):21839-21848

20. Galán-Arriola C, Villena-Gutiérrez R, Higuero-Verdejo MI, Díaz-Rengifo IA, Pizarro G, López GJ, Molina-Iracheta Ad, Pérez-Martínez C, García RD, González-Calle D, Lobo M, Sánchez PL, Oliver E, Córdoba R, Fuster V, Sánchez-González J, Ibanez B (2020) Remote ischaemic preconditioning ameliorates anthracycline-induced cardiotoxicity and preserves mitochondrial integrity. Cardiovascular research 117 (4):1132-1143. doi:10.1093/cvr/cvaa181

21. Goetzenich A, Hatam N, Zernecke A, Weber C, Czarnotta T, Autschbach R, Christiansen S (2009) Alteration of matrix metalloproteinases in selective left ventricular adriamycin-induced cardiomyopathy in the pig. The Journal of heart and lung transplantation : the official publication of the International Society for Heart Transplantation 28 (10):1087-1093. doi:10.1016/j.healun.2009.06.025

22. Gyongyosi M, Lukovic D, Zlabinger K, Spannbauer A, Gugerell A, Pavo N, Traxler D, Pils D, Maurer G, Jakab A, Riesenhuber M, Pircher A, Winkler J, Bergler-Klein J (2019) Liposomal doxorubicin attenuates cardiotoxicity via induction of interferon-related DNA damage resistance. Cardiovascular research. doi:<http://dx.doi.org/10.1093/cvr/cvz192>

23. Adamcova M, Potacova A, Popelova O, Sterba M, Mazurova Y, Aupperle H, Gersl V (2010) Cardiac remodeling and MMPs on the model of chronic daunorubicin-induced cardiomyopathy in rabbits. Physiological research 59 (5):831-836

24. Aupperle H, Garbade J, Schubert A, Barten M, Dhein S, Schoon HA, Mohr FW (2007) Effects of autologous stem cells on immunohistochemical patterns and gene expression of metalloproteinases and their tissue inhibitors in doxorubicin cardiomyopathy in a rabbit model. Veterinary pathology 44 (4):494-503. doi:10.1354/vp.44-4-494

25. Lencova-Popelova O, Jirkovsky E, Mazurova Y, Lenco J, Adamcova M, Simunek T, Gersl V, Sterba M (2014) Molecular remodeling of left and right ventricular myocardium in chronic anthracycline cardiotoxicity and post-treatment follow up. PloS one 9 (5):e96055. doi:10.1371/journal.pone.0096055

26. Potacova A, Adamcova M, Sterba M, Popelova O, Simunek T, Mazurova Y, Guncova I, Gersl V (2007) A pilot study of matrix metalloproteinases on the model of daunorubicin-induced cardiomyopathy in rabbits. Acta Medica (Hradec Kralove) 50 (2):109-111

27. Rodrigues PG, Miranda-Silva D, Costa SM, Barros C, Hamdani N, Moura C, Mendes MJ, Sousa-Mendes C, Trindade F, Fontoura D, Vitorino R, Linke WA, Leite-Moreira AF, Falcao-Pires I (2019) Early myocardial changes induced by doxorubicin in the nonfailing dilated ventricle. American Journal of Physiology - Heart and Circulatory Physiology 316 (3):H459-H475. doi:<http://dx.doi.org/10.1152/ajpheart.00401.2018>

28. Sterba M, Popelova O, Lenco J, Fucikova A, Brcakova E, Mazurova Y, Jirkovsky E, Simunek T, Adamcova M, Micuda S, Stulik J, Gersl V (2011) Proteomic insights into chronic anthracycline cardiotoxicity. Journal of molecular and cellular cardiology 50 (5):849-862. doi:10.1016/j.yjmcc.2011.01.018

29. Abu Gazia M, El-Magd MA (2018) Ameliorative Effect of Cardamom Aqueous Extract on Doxorubicin-Induced Cardiotoxicity in Rats. Cells, tissues, organs 206 (1-2):62-72. doi:10.1159/000496109

30. Arafa MH, Mohammad NS, Atteia HH, Abd-Elaziz HR (2014) Protective effect of resveratrol against doxorubicin-induced cardiac toxicity and fibrosis in male experimental rats. J Physiol Biochem 70 (3):701-711. doi:10.1007/s13105-014-0339-y

31. Arozal W, Watanabe K, Veeraveedu PT, Ma M, Thandavarayan RA, Sukumaran V, Suzuki K, Kodama M, Aizawa Y (2010) Protective effect of carvedilol on daunorubicin-induced cardiotoxicity and nephrotoxicity in rats. Toxicology 274 (1-3):18-26. doi:10.1016/j.tox.2010.05.003

32. Arozal W, Watanabe K, Veeraveedu PT, Thandavarayan RA, Harima M, Sukumaran V, Suzuki K, Kodama M, Aizawa Y (2010) Effect of telmisartan in limiting the cardiotoxic effect of daunorubicin in rats. The Journal of pharmacy and pharmacology 62 (12):1776-1783. doi:10.1111/j.2042-7158.2010.01196.x

33. Bartekova M, Simoncikova P, Fogarassyova M, Ivanova M, Okruhlicova L, Tribulova N, Dovinova I, Barancik M (2015) Quercetin improves postischemic recovery of heart function in doxorubicin-treated rats and prevents doxorubicin-induced matrix metalloproteinase-2 activation and apoptosis induction. International journal of molecular sciences 16 (4):8168-8185. doi:10.3390/ijms16048168

34. Cappetta D, Esposito G, Piegari E, Russo R, Ciuffreda LP, Rivellino A, Berrino L, Rossi F, De Angelis A, Urbanek K (2016) SIRT1 activation attenuates diastolic dysfunction by reducing cardiac fibrosis in a model of anthracycline cardiomyopathy. International journal of cardiology 205:99-110. doi:10.1016/j.ijcard.2015.12.008

35. Cappetta D, Esposito G, Coppini R, Piegari E, Russo R, Ciuffreda LP, Rivellino A, Santini L, Rafaniello C, Scavone C, Rossi F, Berrino L, Urbanek K, De Angelis A (2017) Effects of ranolazine in a model of doxorubicin-induced left ventricle diastolic dysfunction. British journal of pharmacology 174 (21):3696-3712. doi:<http://dx.doi.org/10.1111/bph.13791>

36. Chan KY, Xiang P, Zhou L, Li K, Ng PC, Wang CC, Zhang L, Deng HY, Pong NH, Zhao H, Chan WY, Sung RY (2011) Thrombopoietin protects against doxorubicin-induced cardiomyopathy, improves cardiac function, and reversely alters specific signalling networks. European journal of heart failure 13 (4):366-376. doi:10.1093/eurjhf/hfr001

37. Chen X, Guo Z, Wang P, Xu M (2014) Erythropoietin modulates imbalance of matrix metalloproteinase-2 and tissue inhibitor of metalloproteinase-2 in doxorubicin-induced cardiotoxicity. Heart, lung & circulation 23 (8):772-777. doi:10.1016/j.hlc.2014.02.015

38. Chen PY, Hou CW, Shibu MA, Day CH, Pai P, Liu ZR, Lin TY, Viswanadha VP, Kuo CH, Huang CY (2017) Protective effect of Co-enzyme Q10 On doxorubicin-induced cardiomyopathy of rat hearts. Environmental toxicology 32 (2):679-689. doi:10.1002/tox.22270

39. Chua S, Lee FY, Chiang HJ, Chen KH, Lu HI, Chen YT, Yang CC, Lin KC, Chen YL, Kao GS, Chen CH, Chang HW, Yip HK (2016) The cardioprotective effect of melatonin and exendin-4 treatment in a rat model of cardiorenal syndrome. Journal of pineal research 61 (4):438-456. doi:10.1111/jpi.12357

40. Das J, Ghosh J, Manna P, Sil PC (2011) Taurine suppresses doxorubicin-triggered oxidative stress and cardiac apoptosis in rat via up-regulation of PI3-K/Akt and inhibition of p53, p38-JNK. Biochemical Pharmacology 81 (7):891-909. doi:<http://dx.doi.org/10.1016/j.bcp.2011.01.008>

41. El-Said NT, Mohamed EA, Taha RA (2019) Irbesartan suppresses cardiac toxicity induced by doxorubicin via regulating the p38-MAPK/NF-kappaB and TGF-beta1 pathways. Naunyn-Schmiedeberg's archives of pharmacology 392 (6):647-658. doi:10.1007/s00210-019-01624-3

42. Gordiienko Iu A, Babets YV, Kulinich AO, Shevtsova AI, Ushakova GO (2014) Activity of trypsin-like enzymes and gelatinases in rats with doxorubicin cardiomyopathy. Ukrainian biochemical journal 86 (6):139-146

43. Hang P, Zhao J, Sun L, Li M, Han Y, Du Z, Li Y (2017) Brain-derived neurotrophic factor attenuates doxorubicin-induced cardiac dysfunction through activating Akt signalling in rats. Journal of cellular and molecular medicine 21 (4):685-696. doi:10.1111/jcmm.13012

44. Hong YM, Lee H, Cho MS, Kim KC (2017) Apoptosis and remodeling in adriamycin-induced cardiomyopathy rat model. Korean journal of pediatrics 60 (11):365-372. doi:10.3345/kjp.2017.60.11.365

45. Ivanova M, Dovinova I, Okruhlicova L, Tribulova N, Simoncikova P, Barte-Kova M, Vlkovicova J, Barancik M (2012) Chronic cardiotoxicity of doxorubicin involves activation of myocardial and circulating matrix metalloproteinases in rats. Acta pharmacologica Sinica 33 (4):459-469. doi:<http://dx.doi.org/10.1038/aps.2011.194>

46. Levick SP, Soto-Pantoja DR, Bi J, Hundley WG, Widiapradja A, Manteufel EJ, Bradshaw TW, Melendez GC (2018) Doxorubicin-Induced Myocardial Fibrosis Involves the Neurokinin-1 Receptor and Direct Effects on Cardiac Fibroblasts. Heart, lung & circulation. doi:10.1016/j.hlc.2018.08.003

47. Lim SC (2013) Interrelation between Expression of ADAM 10 and MMP 9 and Synthesis of Peroxynitrite in Doxorubicin Induced Cardiomyopathy. Biomolecules & therapeutics 21 (5):371-380. doi:10.4062/biomolther.2013.034

48. Liu G, Liu Y, Wang R, Hou T, Chen C, Zheng S, Dong Z (2016) Spironolactone Attenuates Doxorubicin-induced Cardiotoxicity in Rats. Cardiovascular therapeutics 34 (4):216-224. doi:10.1111/1755-5922.12189

49. Lou H, Danelisen I, Singal PK (2005) Involvement of mitogen-activated protein kinases in adriamycin-induced cardiomyopathy. American journal of physiology Heart and circulatory physiology 288 (4):H1925-1930. doi:10.1152/ajpheart.01054.2004

50. Mantawy EM, Esmat A, El-Bakly WM, Salah ElDin RA, El-Demerdash E (2017) Mechanistic clues to the protective effect of chrysin against doxorubicin-induced cardiomyopathy: Plausible roles of p53, MAPK and AKT pathways. Scientific reports 7 (1):4795. doi:10.1038/s41598-017-05005-9

51. Medeiros-Lima DJM, Carvalho JJ, Tibirica E, Borges JP, Matsuura C (2019) Time course of cardiomyopathy induced by doxorubicin in rats. Pharmacological reports : PR 71 (4):583-590. doi:10.1016/j.pharep.2019.02.013

52. Mohamed EA, Kassem HH (2018) Protective effect of nebivolol on doxorubicin-induced cardiotoxicity in rats. Archives of medical science : AMS 14 (6):1450-1458. doi:10.5114/aoms.2018.79008

53. Pandey S, Kuo WW, Ho TJ, Yeh YL, Shen CY, Chen RJ, Chang RL, Pai PY, Padma VV, Huang CY, Huang CY (2019) Upregulation of IGF-IIRalpha intensifies doxorubicin-induced cardiac damage. Journal of cellular biochemistry. doi:10.1002/jcb.28957

54. Richard C, Ghibu S, Delemasure-Chalumeau S, Guilland JC, Des Rosiers C, Zeller M, Cottin Y, Rochette L, Vergely C (2011) Oxidative stress and myocardial gene alterations associated with Doxorubicin-induced cardiotoxicity in rats persist for 2 months after treatment cessation. The Journal of pharmacology and experimental therapeutics 339 (3):807-814. doi:10.1124/jpet.111.185892

55. Shaker O, Sourour DA (2010) How to protect doxorubicin-induced cardiomyopathy in male albino rats? Journal of cardiovascular pharmacology 55 (3):262-268. doi:10.1097/FJC.0b013e3181cf91ac

56. Shati AA, El-Kott AF (2019) Acylated ghrelin prevents doxorubicin-induced cardiac intrinsic cell death and fibrosis in rats by restoring IL-6/JAK2/STAT3 signaling pathway and inhibition of STAT1. Naunyn-Schmiedeberg's archives of pharmacology. doi:10.1007/s00210-019-01664-9

57. Sun R, Wang J, Zheng Y, Li X, Xie T, Li R, Liu M, Cao Y, Lu L, Zhang Q, Zhang P (2017) Traditional Chinese medicine baoxin decoction improves cardiac fibrosis of rats with dilated cardiomyopathy. Experimental and therapeutic medicine 13 (5):1900-1906. doi:10.3892/etm.2017.4223

58. Tian XQ, Ni XW, Xu HL, Zheng L, ZhuGe DL, Chen B, Lu CT, Yuan JJ, Zhao YZ (2017) Prevention of doxorubicin-induced cardiomyopathy using targeted MaFGF mediated by nanoparticles combined with ultrasound-targeted MB destruction. International journal of nanomedicine 12:7103-7119. doi:10.2147/ijn.S145799

59. Vacchi-Suzzi C, Bauer Y, Berridge BR, Bongiovanni S, Gerrish K, Hamadeh HK, Letzkus M, Lyon J, Moggs J, Paules RS, Pognan F, Staedtler F, Vidgeon-Hart MP, Grenet O, Couttet P (2012) Perturbation of microRNAs in rat heart during chronic doxorubicin treatment. PloS one 7 (7):e40395. doi:10.1371/journal.pone.0040395

60. Wergeland A, Bester DJ, Sishi BJ, Engelbrecht AM, Jonassen AK, Van Rooyen J (2011) Dietary red palm oil protects the heart against the cytotoxic effects of anthracycline. Cell biochemistry and function 29 (5):356-364. doi:10.1002/cbf.1756

61. Wu X, Qi X, Lu Y, Lin C, Yuan Y, Zhu Q, Yin Q, Li W, Li Y, Bian H (2016) Liguzinediol protects against cardiac fibrosis in rats in vivo and in vitro. Biomedicine & pharmacotherapy = Biomedecine & pharmacotherapie 80:260-267. doi:10.1016/j.biopha.2016.03.033

62. Xiang P, Deng HY, Li K, Huang GY, Chen Y, Tu L, Ng PC, Pong NH, Zhao H, Zhang L, Sung RY (2009) Dexrazoxane protects against doxorubicin-induced cardiomyopathy: upregulation of Akt and Erk phosphorylation in a rat model. Cancer Chemother Pharmacol 63 (2):343-349. doi:10.1007/s00280-008-0744-4

63. Xiao J, Sun GB, Sun B, Wu Y, He L, Wang X, Chen RC, Cao L, Ren XY, Sun XB (2012) Kaempferol protects against doxorubicin-induced cardiotoxicity in vivo and in vitro. Toxicology 292 (1):53-62. doi:10.1016/j.tox.2011.11.018

64. Yu X, Cui L, Zhang Z, Zhao Q, Li S (2013) alpha-Linolenic acid attenuates doxorubicin-induced cardiotoxicity in rats through suppression of oxidative stress and apoptosis. Acta biochimica et biophysica Sinica 45 (10):817-826. doi:10.1093/abbs/gmt082

65. Yu SY, Liu L, Li P, Li J (2013) Rapamycin inhibits the mTOR/p70S6K pathway and attenuates cardiac fibrosis in adriamycin-induced dilated cardiomyopathy. The Thoracic and cardiovascular surgeon 61 (3):223-228. doi:10.1055/s-0032-1311548

66. Yu Q, Li Q, Na R, Li X, Liu B, Meng L, Liutong H, Fang W, Zhu N, Zheng X (2014) Impact of repeated intravenous bone marrow mesenchymal stem cells infusion on myocardial collagen network remodeling in a rat model of doxorubicin-induced dilated cardiomyopathy. Molecular and cellular biochemistry 387 (1-2):279-285. doi:10.1007/s11010-013-1894-1

67. Zhang CJ, Huang Y, Lu JD, Lin J, Ge ZR, Huang H (2018) Upregulated microRNA-132 rescues cardiac fibrosis and restores cardiocyte proliferation in dilated cardiomyopathy through the phosphatase and tensin homolog-mediated PI3K/Akt signal transduction pathway. Journal of cellular biochemistry. doi:10.1002/jcb.27081

68. Zhang K, Zhang J, Wang X, Wang L, Pugliese M, Passantino A, Li J (2018) Cardioprotection of Sheng Mai Yin a classic formula on adriamycin induced myocardial injury in Wistar rats. Phytomedicine : international journal of phytotherapy and phytopharmacology 38:1-11. doi:10.1016/j.phymed.2017.09.001
